# The effects of *Pseudomonas* strains isolated from *Achnatherum inebrians* on plant growth: A genomic perspective

**DOI:** 10.1111/1758-2229.70011

**Published:** 2024-10-10

**Authors:** Jinjin Liang, Bowen Liu, Michael J. Christensen, Chunjie Li, Xingxu Zhang, Zhibiao Nan

**Affiliations:** ^1^ State Key Laboratory of Herbage Improvement and Grassland Agroecosystems, Center for Grassland Microbiome, Key Laboratory of Grassland Livestock Industry Innovation, Ministry of Agriculture and Rural Affairs College of Pastoral Agriculture Science and Technology, Lanzhou University Lanzhou China; ^2^ Grasslands Research Centre, AgResearch Palmerston North New Zealand

## Abstract

*Achnatherum inebrians* is a perennial grass widely distributed in northwest China. Nearly all wild *A. inebrians* plants are infected by *Epichloë* endophytes. In this study, bacteria from the phyllosphere were isolated from leaves of both endophyte‐free and endophyte‐infected *A. inebrians* and sequenced for identification. *Pseudomonas*, comprising 48.12% of the culturable bacterial communities, was the most dominant bacterial genus. Thirty‐four strains from 12 *Pseudomonas* species were used to inoculate *A. inebrians* seeds and plants. Results indicated that *Epichloë* significantly increased the diversity and richness index of the phyllosphere. *Pseudomonas* Sp1, Sp3, Sp5 and Sp7 had a significantly positive effect on plant growth and photosynthesis, whereas Sp10, Sp11 and Sp12 had a significantly negative effect. Whole‐genome and pan‐genome analysis suggested that the variability in the effects of *Pseudomonas* on *A. inebrians* was related to differences in genome composition and genomic islands.

## Graphical abstract

*Achnatherum inebrians*, a perennial grass in northwest China, is almost entirely infected by *Epichloë* endophytes. This study isolated and sequenced phyllosphere bacteria from both endophyte‐free and endophyte‐infected *A. inebrians*. *Pseudomonas*, the dominant genus (48.12%), showed varied effects on plant growth and photosynthesis, with some strains positively influencing these factors and others negatively. Whole‐genome and pan‐genome analysis revealed that these effects were linked to differences in genome composition and genomic islands.

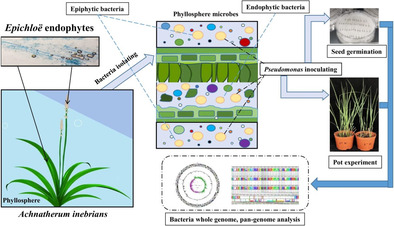

## INTRODUCTION

Rhizosphere microbes not only promote the growth and development of host plants (Trivedi et al., 2020), but they also function as a second genome of plants (Feng et al., 2023). As the most beneficial rhizosphere microorganisms for host plants, plant growth‐promoting rhizobacteria (PGPR) have the potential to directly promote plant growth and inhibit plant pathogens (Lugtenberg & Kamilova, 2009). PGPR include many bacterial genera such as *Azotobacter*, *Bacillus*, *Burkholderia*, *Enterobacter*, *Rhizobium* and *Pseudomonas* (Backer et al., 2018); among them, *Pseudomonas* has received extensive attention (Chiappero et al., 2019; Dimkic et al., 2022), mainly because *Pseudomonas* bacteria are the dominant rhizosphere growth‐promoting bacteria that are known to play an essential role in crops and plants.

*Pseudomonas* belongs to Pseudomonadota, Pseudomonadales and Pseudomonadaceae. This genus occupies a wide ecological niche in the ecosystem (Gomila et al., 2015). In nature, most *Pseudomonas* strains have harmful effects on humans and animals, for example, *Pseudomonas aeruginosa*, *Pseudomonas fluorescens* and *Pseudomonas maltophilia* (Parra‐Sánchez et al., 2023). But *Pseudomonas* bacteria isolated from plants mostly had a positive influence on plant growth and development, such as *Pseudomonas* strains isolated from rice significantly inhibited fungal disease to promote rich fitness (Yang et al., 2023); *Pseudomonas* bacteria can dissolve phosphate, which may be critical in phosphorus‐deficient agricultural soils to increase plant yield by enhancing phosphorus acquisition and distribution of plants (Liu et al., 2024). *Pseudomonas* bacteria not only individually promote plant growth, but systematically change the communities of wheat soil PGPR to increase plant growth (Garrido‐Sanz et al., 2023). Therefore, *Pseudomonas* bacteria play an important role in promoting plant growth and development.

Endophytic fungi of the genus *Epichloë* are present in all tissues, apart from the roots, of many cool‐season grasses, and for the majority of *Epichloë*—grass associations transmission is entirely vertical, in seeds of the host grass. For *Epichloë* grass associations in which transmission is entirely vertical, host plants are symptomless, and the growth of the fungus and the host plant is synchronized (Christensen et al., 2008). As a perennial grass, *Achnatherum inebrians* is widely distributed in Gansu, Qinghai, Neimeng and Xinjiang provinces. *Epichloë* endophytes after having found that nearly 100% of *A. inebrians* plants in these natural grasslands were host to an *Epichloë* endophyte (Nan & Li, 2000). The presence of *Epichloë* endophytes enhances the resistance of *A. inebrians* to diseases (Kou et al., 2021) and insect pests (Zhang et al., 2012), as well as to abiotic stresses such as drought (Zhong et al., 2022), salinity (Wang et al., 2018) and low temperature (Chen et al., 2016). Meanwhile, *Epichloë* endophytes are the main cause of animal poisoning caused by *A. inebrians*, giving rise to its common name of drunken horse grass (Liang et al., 2017; Zhang et al., 2014); *A. inebrians* plants that are not host to these endophytic fungi can be eaten by livestock and herbivores, and so has the potential as an important forage grass (Nan et al., 2021).

Previous studies of our team found that *Epichloë* endophytes had a significant influence on rhizosphere soil (Ju et al., 2020), phyllosphere (Liu et al., 2022) and seedborne (Liang et al., 2023) bacterial communities through the application of high‐throughput sequencing technology, further research concerned isolated bacteria from *A. inebrians* root and rhizosphere, found that *Pseudomonas* was one of the most dominated genera of *A. inebrians* (Ju et al., 2021). *Pseudomonas* is a dominant bacterial genus widely distributed in the rhizosphere, seeds and leaves, and its presence may be closely related to the seed germination, seedling growth and persistence of host plants. The study found that the presence of an *Epichloë* endophyte increased the abundance of *Pseudomonas* associated with *Lolium multiflorum* (Bastías et al., 2021), and this along with Ju et al. (2021) strongly indicated that the effect of *Epichloë* endophytes and *Pseudomonas* on promoting the growth of grasses and its molecular mechanism needs to be further clarified.

In this study, bacteria were isolated from endophyte‐infected (EI) and endophyte‐free (EF) *A. inebrians* leaves, and those identified as *Pseudomonas* were retained. Selected isolates were used to inoculate seeds and seedlings of *A. inebrians* to determine their ability to enhance seed germination and subsequent growth of seedlings. The selected isolates were subjected to genetic analysis to examine diversity within *Pseudomonas* associated with *A. inebrians*. This study aimed to assess: (1) if the presence of an *Epichloë* endophyte would change the composition and diversity of the phyllosphere culturable bacteria of *A. inebrians*. (2) If there are diversity effects among *Pseudomonas* isolates on seed germination and seedling growth of *A. inebrians*. (3) If diverse effects of *Pseudomonas* on *A. inebrians* were related to genomic composition and specific genomic islands.

## EXPERIMENTAL PROCEDURES

### *Isolation, cultivation and identification of bacteria in* A. inebrians

#### A. inebrians *collection and experimental site*

EI and EF *A. inebrians* plants were collected from an experimental field in the Yuzhong campus (104°399 E, 35°899 N, altitude, 1653 m) of the College of Pastoral Agriculture Science and Technology of Lanzhou University. These EI and EF plots, also used in other studies, were established in 2017 and managed as described by Liu et al. (2022) and Liang et al. (2023).

Leaf samples were collected from three plots which were randomly selected, and EI and EF samples were obtained from two sub‐plots of each plot. The method of 5‐point sampling was used to obtain the leaves of *A. inebrians* plants. The third or fourth newest leaf of a tiller was selected from the plants in five sampling sites of each sub‐plot, above leaves were mixed, and each mixed leaves sample was one replicate for isolating the phyllosphere bacteria.

#### Bacteria isolation

A total of 3 g of fresh leaf samples was placed in 50 mL conical bottles were gently washed with sterile water to remove soil and impurities. About 30 mL of sterile phosphate‐buffered saline (PBS) solution (10^−1^) was placed into each 50 mL conical bottle and sealed with a sealing film. After shaking at 150 r/min shake for 3 h, the flasks were allowed to stand for 5 min, and then a dilution gradient (10^−2^ to 10^−7^) was created using sterile water and this was used for the isolation of *A. inebrians* epiphytic bacteria. The above leaves were surface sterilized in 75% ethanol for 1 min, 1% sodium hypochlorite for 2 min and 75% ethanol for 30 s, and then washed three times in sterile water; these sterilized leaves were used for the isolation *A. inebrians* endophyte bacteria (Liu et al., 2022). Subsequently, the treated leaves were placed in a mortar, and 30 mL of sterile PBS (10^−1^) was added prior to grinding, and filtering. The filtrate was diluted (10^−2^ to 10^−7^) with sterile water for the isolation and culture of endophytic bacteria. Three replications of 100 μL of the above‐diluted filtrate were coated on R_2_A agar (Gibbs & Hayes, 1988; Ju et al., 2021), and these were placed in an inverted incubator (DHP9082A, Shanghai) at 25 ± 2°C for 24 h.

#### DNA extraction and PCR amplification

The DNA of all the bacterial isolates was extracted with an Ezup Column Bacterial Genomic DNA Purification kit (Tiangen, Beijing) that used the bacteria universal primers 27F (5′‐AGAGTTTGATCMTGGCTCAG‐3′) and 1492R (5′‐GGTTACCTTGTTACGACTT‐3′) for amplification for the gene sequences. The following cycle conditions were used for PCR amplification (25 μL system including 1 μL upper primer, 1 μL lower primer, 1 μL template (DNA), 12.5 μL Tap polymerase (Sangon Biotech, Shanghai), 9.5 μL dd H_2_O): 95°C for 300 s, 30 cycles (95°C for 60 s; 58°C for 60 s; 72°C for 60 s and 72°C for 120 s), and 72°C for 10 min (Tan et al., 2017). PCR product samples were stored in a 4°C refrigerator and later bacterial sequence detection was conducted via the Sangon Biotech (Shanghai, China).

#### Strain identification

The 16S rRNA gene sequences were submitted and checked in the NCBI database (https://www.ncbi.nlm.nih.gov/). The NJ (neighbour‐joining) method of MEGA software (v7.0.26) was used to construct the sequences of culturable bacteria of *A. inebrians*. The phylogenetic tree involved in the study was referenced by Kumar et al. (2018).

#### Alpha and beta diversity analysis

Alpha diversity was assessed by utilizing Shannon (https://mothur.org/wiki/shannon/), Simpson (https://mothur.org/wiki/simpson/), Chao (https://mothur.org/wiki/chao/) and ACE index (https://mothur.org/wiki/ace/) via Mother software (v.1.30).

Principal coordinate analysis (PCoA) and the statistically significant differences of endophytic and epiphytic bacterial communities of EI and EF *A. inebrians* leaves were tested through permutation multivariate analysis of variance (PERMANOVA) and analysis of similarity (ANOSIM) based on the Bray Curtis dissimilarities using the vegan package in R (v.4.0.3).

#### Germination experiment, conducted in petri dishes

We chose the most dominant genera *Pseudomonas* of the phyllosphere bacteria of *A. inebrians* to do the further inoculating experiment. Twelve species, 34 strains, of *Pseudomonas* were involved in this study. Detailed information on these *Pseudomonas* strains is shown in the following Table 1.

**TABLE 1:** *Pseudomonas* corresponding to the serial number used in the study.

*Pseudomonas*	Strain	Species
*Pseudomonas putida*	St1, St2, St3, St4, St5, St6, St7, St8, St9, St10, St11, St12, St13, St14, St15	Sp1
*Pseudomonas alkylphenolica*	St16	Sp2
*Pseudomonas oryzihabitans*	St17, St18, St19, St20	Sp3
*Pseudomonas japonica*	St21	Sp4
*Pseudomonas* sp. TF6	St22, St23	Sp5
*Pseudomonas* sp. TBzl018	St24, St25	Sp6
*Stutzerimonas stutzeri* (formerly known as *Pseudomonas stutzeri*)	St26, St27, St28	Sp7
*Pseudomonas oleovorans* subsp. *Oleovans*	St29	Sp8
*Pseudomonas* sp. Vsos‐36	St30	Sp9
*Pseudononas koreensis*	St31	Sp10
*Pseudomonas parafulva*	St32, St33	Sp11
*Pseudomonas fulva*	St34	Sp12

Each *Pseudomonas* strain was used to prepare bacterial suspensions that were decanted into sterilized centrifugal tubes. LB liquid medium (5 g/L yeast powder, 5 g/L NaCl, 10 g/L tryptone) was used in the germination experiment. Each isolated and purified bacterial colony was added to a liquid medium and placed in a constant temperature culture shaker (TS‐2102C, Shanghai Jechen). The rotation speed of the shaker was 180 r/min and the temperature was 28 ± 2°C, for 48 h. The concentration of bacterial solutions was OD_600_ = 0.9 as determined by a spectrometer; meanwhile, we tested the purity of the bacterial solution to ensure that there was no pollution in the bacterial solution via the nucleic acid detector (NanoDrop 1000, Germany).

Each *Pseudomonas* bacteria solution was added into a sterilized 5 mL centrifuge tube, and then the surface‐sterilized EI/EF seeds (75% ethanol for 2 min and 1% sodium hypochlorite for 5 min) were soaked in the bacteria solution (lysogeny broth [LB] liquid medium, soaked as the control), for 5 min. The seeds were then removed and placed in a petri dish (90 mm in diameter) containing two layers of sterile filter paper. Sterile distilled water was dripped onto the sterile filter paper until a water film had formed on the surface of the filter paper before the seeds were spread evenly on the filter paper to calculate the germination rate. Four biological replications with each petri dish containing 50 *A. inbrians* seeds were carried out. The Petri dishes were placed in a constant temperature incubator (DHP9082A, Shanghai), with a daily 12 h of light and darkness; the temperature was 25 ± 2°C, and the relative humidity was 70 ± 10%. Five dishes were replicated in each group. Sterile distilled water was added every 24 h to ensure that the moisture content of filter paper in the dishes was maintained (Ju et al., 2021).

#### Indexes of the germination experiment

Germination percentage (GP), GP = (Gt/*T*) × 100%, where *T* is the total number of seeds, and Gt is the number of germinated seeds on the last day of germination.

Germination potential for seed germination peak; namely, percentage of seed that germinated after 3 days.

Germination index (GI), GI = ∑(Gt/Dt), the Gt for germination test period daily germination numbers, Dt for germination days, ∑ for combined.

Root length, average root length of germinating seeds in every dish;

Seeding length of germinating seeds for each dish to obtain average seedling growth;

Fresh weight of germinating seeds for each dish to obtain total fresh weight;

The dry weight of germinating seeds for each plate to obtain the total dry weight.

A comprehensive evaluation of the growth‐promoting effect: The membership function method was used to evaluate the growth‐promoting effect by synthesizing seven germination indexes (Zou et al., 2024), the formula is:

Positive correlation:

$$U_{\mathrm{ij}}=\frac{X_{\mathrm{ij}}-X_{j\min}}{X_{j\max}-X_{j\min}}$$

Negative correlation:

$$U_{\mathrm{ij}}=1-\frac{X_{\mathrm{ij}}-XX_{j\;\min}}{X_{j\max}-X_{j\min}}$$

In these formulas, *U*_*ij*_ represents the growth promotion membership function value of the *j* index of type *i*, *X*_*ij*_ represents the measured value of the *j* index of type *i*, *i* represents *Pseudomonas* inoculum *j* represents the germination index of *A. inebrians* seeds, *X*_*j*max_ represents the maximum value of *j* index of all kinds, and *X*_*j*min_ represents the minimum value of *j* index of all kinds. According to the above formula, the membership degree of each processing seven germination indexes was calculated and then the same processing of 7 average germination index of the membership degree is used; the growth promotion of *Pseudomonas* can be divided into five levels according to the average membership degree: *U*_*ij*_ (0.60–1.00) was Promotion, *U*_*i*j_ (0.30–0.59) was no effect (N); *U*_*ij*_ (0–0.29) was Inhibition.

#### Greenhouse pot experiment design

EI and EF seeds were randomly selected. The seeds were surface sterilized with 75% alcohol for 2 min, then with 1% sodium hypochlorite solution for 5 min, and finally washed four to five times with sterile distilled water. The seeds were planted in sterilized plastic pots (upper diameter 10.3 cm, lower diameter 8 cm) containing 90 g of sterilized vermiculite mixed 2:1 with black soil (treated at 120°C for 5 h in autoclave). 13 cm, including 56 EF and 56 EI treatment pots, were placed in the controlled environment greenhouse of Lanzhou University (temperature: 25 ± 2°C; Humidity: 46 ± 2%). 3 EF or EI seeds were sown in each pot. After the second true leaf of the *A. inebrians* seedlings grew, 50 mL of half‐strength Hoagland nutrient solution was added every second day (Xia et al., 2018). The placement of pots was regularly randomly adjusted, and water was added daily to each pot to retain the saturated soil water content.

In this experiment, *A. inebrians* seedlings with (EI) or without (EF) the *Epichloë* endophyte were inoculated with one of the 12 *Pseudomonas* strains of the 12 different species (Sp1, Sp2, Sp3, Sp4, Sp5, Sp5, Sp7, Sp8, Sp9, Sp10, Sp11 and Sp12), and the LB medium control (LBCK) were inoculated with LB liquid medium control. As the main factor, the water control (WCK) was inoculated with sterile distilled water. According to the method of He et al. (2018), 2‐month‐old EI and EF seedlings were inoculated into the root system, by using a sterile pipette, with 10 mL *Pseudomonas* bacteria solution, with the concentration of bacteria solution was OD_600_ = 0.9, while 10 mL sterilized LB nutrient solution or 10 mL sterile distilled water was used as the control. Inoculation was repeated 1 and 2 weeks after the first inoculation to ensure that *Pseudomonas* could colonize the roots of the plants. After each inoculation, all the plants were placed in a greenhouse (temperature: 18–20°C; Humidity: 70%; Light: 1500 μmol × m^−2^ × s^−1^, the ratio of light to darkness was 16 h:8 h). The number of plants with developing inflorescences was recorded from the 25th day, and all the growth indexes were measured on the 45th day.

#### Indexes of greenhouse pots experiment

The index of plant height was based on the height of the tip of the longest leaf of *A. inebrians* plants of each pot above the potting mix; the total tiller number and number of reproductive tillers were counted to obtain the tiller number per *A. inebrians* plant.

The ground/underground biomass measurement: Forty‐five days after seedlings were inoculated with bacteria, the *A. inebrian* plants were removed from the pots and the roots were washed to remove the potting mixture, taking care to maintain the integrity of the root system. After the washing, the plants were dried on absorbent paper, following which the roots were excised and the two portions of each plant were weighed to obtain the fresh weight values. The average fresh weight of all plants under each treatment was the fresh weight of the plants under this treatment. The root and above‐ground plant samples were placed in an oven at 40°C for 48 h, for dry weight determination.

Photosynthetic index determination: At the end of the experiment, the photosynthetic index and chlorophyll content of all treated EI/EF seedlings were determined. The photosynthetic indexes were determined as in Xia et al. (2018), using a GFS‐3000 portable photo apparatus (WALZ Company, Germany). The light intensity was set at 800 Lx and the temperature was room temperature. The instrument was calibrated every hour. Net photosynthetic rate, transpiration rate, intercellular carbon dioxide concentration and stomatal conductance were measured.

Chlorophyll content determination: The content of chlorophyll was determined using a handheld chlorophyll meter SPAD‐502 plus (Konica Minolta Sensing company, Japan). Leaves with the same photosynthetic index were selected, and the handheld chlorophyll analyzer was used to measure the selected leaves 4 times. The average value of the four readings was the relative value (SPAD value) of the chlorophyll content of this plant. The SPAD value was inserted into the following formula and converted into the specific chlorophyll content of the plant (Ye et al., 2017): *Y* (mg/dm^2^) = 0.0996*X* − 0.152, where *X* was seedling SPAD value and *Y* was specific chlorophyll content of seedlings.

Determination of hormone content in leaves: After 30 days of inoculation with *Pseudomonas*, a total of 50 mg *A. inebrians* leaves were randomly selected from each pot for hormone determination. Indexes of plant hormone, including indole acetic acid (IAA), abscisic acid (ABA), brassinosteroids (BR), gibberellins (GA), cytokinin (CtK), salicylic acid (SA) and jasmonic acid (JA). The fresh *A. inebrians* leaves were frozen in liquid nitrogen for 3–4 h and then stored in a −80°C refrigerator. According to the methods of Kou et al. (2021), the ELISA (enzyme‐linked immunosorbent assays) kit (FANKEL Industrial Co. Ltd., Shanghai, China) was used to determine the content of various hormones, including IAA, ABA, BR, GA, CtK, SA and JA in the leaves of *A. inebrians*.

Determination of nutrient content in leaves: 5 g of dry leaves was used to determine the nutrient content of the leaves. Organic carbon content was determined by the K_2_CrO_7_‐H_2_SO_4_ oxidation method (Nelson & Sommers, 1982). The determination of total nitrogen and total phosphorus content in the leaves was carried out using a fully automated flow injection analyzer (FIAstar 5000 Analyzer, FOSS Analytical, Denmark) (Pan et al., 2015). Flame photometry was used to determine the total potassium and total sodium content of the leaves with reference to the method of Guzman et al. (1992).

#### Bacterial whole‐genome sequencing and pan‐genome analysis

According to the method of Ju et al. (2021), an LB liquid medium was used for culturing *Pseudomonas* strains. The appropriate volume of bacterial solution was transferred to a 2‐mL aseptic centrifuge tube, centrifuged at room temperature of 14,000*g* for 1 min, followed by discarding all the medium. The bacterial precipitation was quickly frozen in liquid nitrogen for 3–4 h, and then transferred to −80°C for preservation, and then transported with dry ice to the Biomarker Technologies Corporation (BMK), Beijing, China, for bacteria whole‐genome sequencing. Strains were sequenced, by referring to the methods adopted by Jiao et al. (2022) and Li et al. (2022), to build a library.

The progress of sequencing data quality control by PacBio HIFI mode, then these gene sequences obtained through the sequencing platform PacBio Sequel II are assembled using Hifiasm (Cheng et al., 2020) software, and cyclized and adjusted by Circlator (v1.5.5) software. Pilon (v1.22) software was used to further correct errors with the second‐generation data, and the genome with higher accuracy was obtained for subsequent analysis.

Analysis of variation and difference among strains: The MUMmer (Delcher et al., 2002) software package was used to compare the genome of each strain with that of the reference species Sp1, and SNPs and small indels were found. In addition, the MUMmer software package was used to compare the genome of each strain with reference species Sp1, and according to the obtained information on homologous collinear regions, the structural variation of each strain was identified and compared with the reference genome. Two kinds of software Alien Hunter (Vernikos & Parkhill, 2006) and GHT‐Finder (Nguyen, Ekstrom, et al., 2015; Nguyen, Schmidt, et al., 2015) were used to predict the horizontal transfer genes of each strain.

Genomic cycle mapping: The genomic information obtained by assembly and prediction, such as tRNA, rRNA, repeat sequence, GC content and gene function information, was mapped by Circos (v0.66) (Krzywinski et al., 2009).

The core and non‐core genes analysis: The software Mugsy (Angiuoli & Salzberg, 2011) was used to compare the genome sequence of all sequenced strains with that of the reference genome. From the comparison results, it was found that the common sequence of all strains was the core genome sequence, and the remaining sequence was the non‐essential genome sequence. Phylogenetic tree: Used the sequencing species of the DNA sequence, and the reference species using IQ TREE (Nguyen, Ekstrom, et al., 2015; Nguyen, Schmidt, et al., 2015)—software to build the evolutionary tree, and this helps the understanding of the evolutionary relationships between test strains.

A total of linear graph: The software Mauve (v2.4.0) was used to conduct collinearity analysis among seven groups of bacterial genomes to find homologous gene and protein sequences, and then the collinearity relationship at the nucleic acid level was obtained according to the position information of homologous genes on the sequence in each bacterium to show the evolutionary relationship between the genomes of different strains (Yang et al., 2021).

Core gene family and non‐core family analysis: OrthoMCL (Li et al., 2003) was used for each strain prediction of protein sequence and the reference genome sequences of proteins to analyse gene families, looking for each strain of Shared (core gene families) and the specific gene families (non‐core).

Gene function prediction: Using KEGG (Kanehisa et al., 2004) (Kyoto Encyclopedia of Genes and Genomes) gene function prediction database and comments.

Gene island prediction: Based on the principle of dinucleotide preference and the presence of at least one mobile gene, the software IslandPath‐DIMOB (v0.2) (Claire & Brinkman, 2018) was used to predict gene islands in the bacterial genomes. All these progress of bacterial gene assembling and data analysis were performed by the platform BMKCloud.

### Statistical analyses

Excel software (Excel v.2010) was used to calculate the results of EI/EF seed germination and plant growing indexes after inoculation with different *Pseudomonas* strains. The data were analysed using SPSS software (SPSS 22.0 version, Chicago, IL, USA) to analyse the effects of inoculation with different *Pseudomonas* strains and the presence of the endophytic fungus on seed germination. The difference in germination potential, germination rate, germination index, root length, seedling length, dry weight and fresh weight was analysed by two‐factor variance analysis. An Independent sample *t* test was used to study whether there were significant differences between EI and EF seed germination indexes, and the significance level was *p* = 0.05. After inoculation with different *P seudomonas* strains, the Duncan test was used to determine whether the difference between the mean values was statistically significant, and the LSD test was used to analyse the significance of differences between all treatments. In all analyses, the 95% confidence level had a statistically significant (*p* < 0.05) effect.

## RESULTS

### *Community composition and diversity of phyllosphere culturable bacteria of* Achnatherum inebrians

**FIGURE 1:**
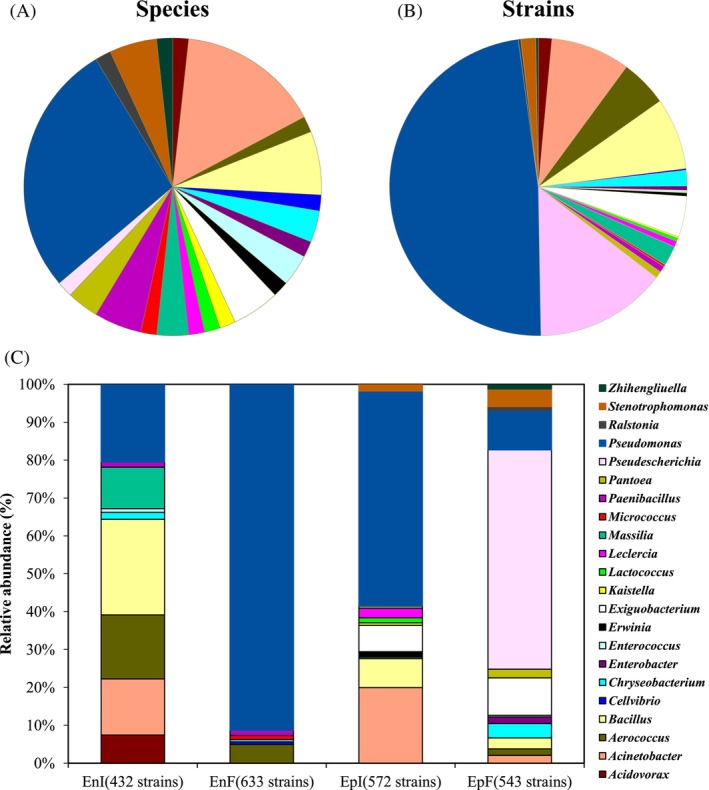
Analysis of community composition of phyllosphere culturable bacteria of *Achnatherum inebrians* at the genus level. (A: The number of species contained in each genus; B: Number of strains contained in each genus; C: Relative abundance of each genus in each treatment. EnI: Endophytic microbes of endophyte‐infected, EnF: Endophytic microbes of endophyte‐free plants, EpI: Epiphytic microbes of endophyte‐infected, EpF: Epiphytic microbes of endophyte‐free plants.)

A total of 2180 bacterial isolates were obtained from the phyllosphere of *Achnatherum inebrians*, and of these, 1065 were endophytic (including 572 EpI strains and 543 EpF strains), and 815 were epiphytic (432 EnI strains and 633 EnF strains) (Figure 1, Table 2). The DNA of all isolates was extracted and 16S rRNA gene sequencing was used to characterize them. The results from the sequencing showed that these bacteria were classified into 4 phyla and 22 genera (Figure 1, Table 2). The community composition of the culturable bacteria of *A. inebrians* leaves mainly comprised Proteobacteria (78.94%), Firmicutes (18.67%), Bacteroidetes (1.70%), Actinobacteria (0.50%) and Flavobacteria (0.18%) at the phylum level (Table 2). *Pseudomonas* (48.12%) was the most dominant bacterial genus obtained from *A. inebrians* leaves and in particular, from EnI and EpI (Figure 1, Table 2). The relative abundance of endophytic *Pseudomonas* was lower in *Epichloë*‐infected leaves (EnI) than in *Epichloë*‐free leaves (EnF), but in contrast, it was more abundant from endophytic of *Epichloë*‐infected *A. inebrians* (EpI) than when the leaves were *Epichloë*‐free (EpF) (Figure 1, Table 2). The next most abundant genus was *Pseudescherichia* (14.31%), which was only within the EpF bacterial community; Massilia (2.16%) and Acidovorax (1.47%) were present only within the EnI community (Figure 1, Table 2). The genus *Paenibacillus* (0.69%) was obtained only from the endophytic bacterial community, whereas *Exiguobacterium* (4.40%) and *Pantoea* (0.73%) were present only in the epiphytic bacterial communities of EpI and EpF (Figure 1, Table 2).

**TABLE 2:** Relative abundance of phyllosphere culturable bacteria of *Achnatherum inebrians*.

Phylum	Genus	Strains number	Relative abundance (genus level) (%)				*F*‐value	*p* Value
			EnI	EnF	EpI	EpF		
Actinobacteria	*Micrococcus*	5	‐	1.10 ± 0.11	‐	‐		
Actinobacteria	*Zhihengliuella*	6	‐	‐	‐	1.18 ± 0.41		
Bacteroidetes	*Chryseobacterium*	37	1.84 ± 0.15 b	0.60 ± 0.30 c	0.37 ± 0.19 c	3.80 ± 0.51 a	24.342	**0.000**
Flavobacteria	*Kaistella*	4	‐	‐	0.66 ± 0.33	‐		
Firmicutes	*Aerococcus*	112	16.9 ± 2.14 a	4.95 ± 0.92 b	‐	1.74 ± 0.76 b	33.900	**0.001**
Firmicutes	*Bacillus*	170	25.27 ± 1.85 a	‐	7.69 ± 0.06 b	2.87 ± 0.44 c	115.627	**0.000**
Firmicutes	*Enterococcus*	6	0.96 ± 0.29	‐	‐	0.40 ± 0.20	2.427	0.194
Firmicutes	*Exiguobacterium*	96	‐	‐	6.97 ± 1.53	9.90 ± 1.31	2.117	0.219
Firmicutes	*Lactococcus*	8	‐	‐	1.33 ± 0.53	‐		
Firmicutes	*Paenibacillus*	15	1.36 ± 0.33	1.36 ± 0.47	‐	‐	0.000	0.996
Proteobacteria	*Acidovorax*	32	7.41 ± 0.26	‐	‐	‐		
Proteobacteria	*Acinetobacter*	188	14.82 ± 0.52 b	‐	19.93 ± 1.67 a	2.04 ± 0.16 c	82.485	**0.000**
Proteobacteria	*Cellvibrio*	4	‐	0.66 ± 0.21	‐	‐		
Proteobacteria	*Enterobacter*	10	‐	‐	‐	1.79 ± 0.31		
Proteobacteria	*Erwinia*	8	‐	‐	1.42 ± 0.22	‐		
Proteobacteria	*Leclercia*	14	‐	‐	2.52 ± 0.54	‐		
Proteobacteria	*Massilia*	47	10.95 ± 0.68	‐	‐	‐		
Proteobacteria	*Pantoea*	16	‐	‐	0.53 ± 0.04 b	2.29 ± 0.58 a	9.273	**0.038**
Proteobacteria	*Pseudescherichia*	312	‐	‐	‐	57.96 ± 2.72		
Proteobacteria	*Pseudomonas*	1049	20.49 ± 1.67 c	91.33 ± 1.36 a	56.83 ± 1.02 b	10.39 ± 0.77 d	974.602	**0.000**
Proteobacteria	*Ralstonia*	6	‐	‐	‐	1.07 ± 0.23		
Proteobacteria	*Stenotrophomonas*	35	‐	‐	1.75 ± 0.10 b	4.57 ± 0.53 a	26.820	**0.007**

*Note*: The significant of alphabets letters were showed in EnI, EnF, EpI and EpF. Bold indicates the significant difference among different treatments. EnI and EnF: endophytic microbes of endophyte‐infected and endophyte‐free plants; EpI and EpF: epiphytic microbes of endophyte‐infected and endophyte‐free plants.

**FIGURE 2:**
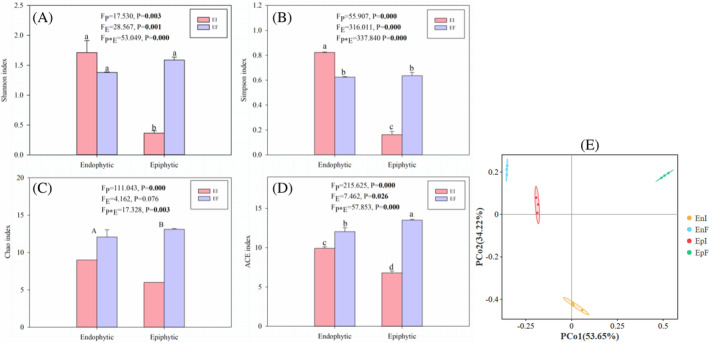
Alpha‐diversity index and beta‐diversity of phyllosphere culturable bacterial communities of *A. inebrians*. Bacterial (A–D) alpha diversity in microbial community, values are mean ± standard error (SEM), with bars indicating SE. Bacteria (E) of principal coordinates analysis (PCoA) based on Bray–Curtis dissimilarities of culturable bacterial communities. (EI: Endophyte‐infected, EF: Endophyte‐free; P: Endophytic and epiphytic environment, E: *Epichloë* endophyte infection status. Different lowercase letters indicate significant (*p* < 0.05) difference between treatments, different capital letters indicate significant (*p* < 0.05) difference between endophytic and epiphytic communities.)

The interaction between the presence of the *Epichloë* endophyte and the treatment of endophytic and epiphytic environment significantly (*p* < 0.05) influenced the Shannon index, Simpson index, and ACE index of the bacterial community (Figure 2A,B,D). The Shannon diversity index of the EnI, EpI and EpF communities was significantly (*p* < 0.05) higher than that of the EnF community (Figure 2A). The Simpson diversity index of the EnI community was significantly (*p* < 0.05) higher than that of the other three communities (Figure 2B). The ACE richness index of the EnI community was significantly (*p* < 0.05) higher than that of the EnF community, while that of the EpI community was lower than that of the EpF community (Figure 2D). Treatment of endophytic and epiphytic environments significantly (*p* < 0.05) affected the Chao1 index of the bacterial community, and the Chao1 index of the epiphytic bacterial communities was significantly (*p* < 0.05) higher than that in endophytic bacterial communities (Figure 2C).

In addition, PCoA showed that both the *Epichloë* endophyte and endophytic and epiphytic environments had a significant influence on the beta‐diversity of the culturable bacterial community of *A. inebrians* (PC1 38.11%, PC2 27.74%) (Figure 2E, Table 3).

**TABLE 3:** The statistical test of similarity (ANOSIM) and permutational multivariate two‐way analysis of variance (PERMANOVA).

Type	Treatment	df	PERMANOVA		ANOSIM	
			*F*	*p*	*R*	*p*
Bacteria	E	1	79.75	**0.0001**	1.000	**0.0104**
	P	1	83.721	**0.0001**	1.000	**0.0099**
	E*P	1	275.66	**0.0001**		

*Note*: Bold indicates the significant difference among different treatments. P: endophytic and epiphytic environment; E: *Epichloë* endophyte infection status.

### *The effect of* Pseudomonas *on seed germination and seeding growth of* A. inebrians

Both the *Epichloë* endophyte and inoculating of *Pseudomonas* had significant (*p* < 0.05) effects on germination potential (*F*_E_ = 29.939, *p* = 0.000; *F*_P_ = 8.838, *p* = 0.000), germination index (*F*_E_ = 28.780, *p* = 0.000; *F*_P_ = 13.348, *p* = 0.000), root length (*F*_E_ = 23.532, *p* = 0.000; *F*_P_ = 16.877, *p* = 0.000), seeding length (*F*_E_ = 91.219, *p* = 0.000; *F*_P_ = 78.436, *p* = 0.000) and fresh weight (*F*_E_ = 89.885, *p* = 0.000; *F*_P_ = 12.907, *p* = 0.000) (Figure 1A,C,D,E). Inoculation of *Pseudomonas* had a significant (*p* < 0.05) influence on the germination percentage of *A. inebrians* seeds (*F*_P_ = 13.802, *p* = 0.000) and on the dry weight of seedlings (*F*_P_ = 40.902, *p* = 0.000) (Figure S1B,G).

**FIGURE 3:**
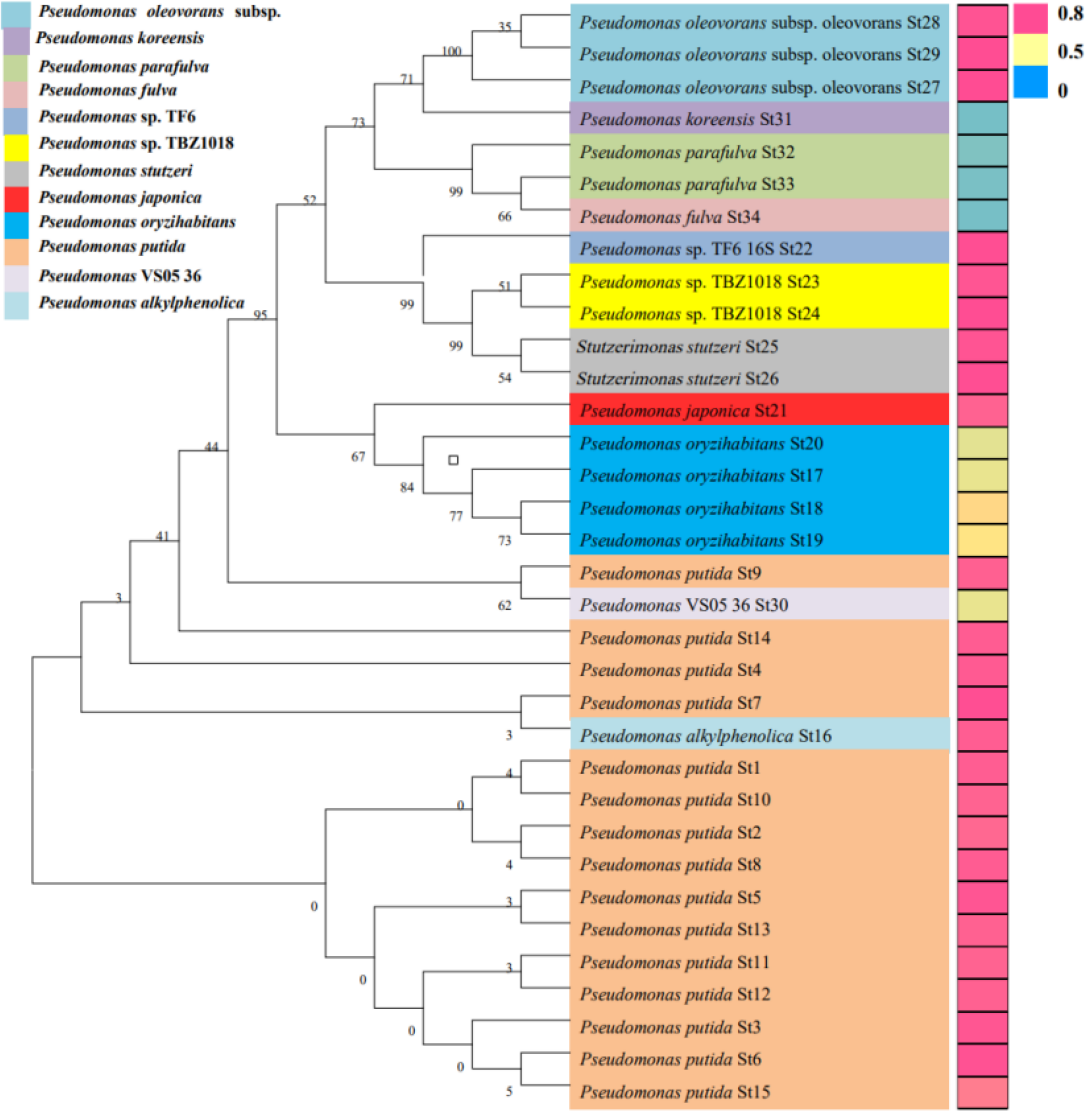
Neighbour‐joining (NJ) phylogenetic tree analysis of *Pseudomonas* bacteria of *A. inebrians*. The neighbour‐joining phylogenetic tree (left) and comprehensive evaluation of 34 *Pseudomonas* strains on *A. inebrians* seeds germination via heat‐map analysis (right). (Number of phylogenetic tree represented evolutionary distance of different bacteria, St1–St34 represented strains of *Pseudomonas*, (0.60–1.00) was Promotion, (0.30–0.59) was no effect (N) and (0–0.29) was Inhibition.

The subordinate function combined with the seven germination indexes of *A. inebrians* was used to comprehensively evaluate the promoter action of 34 *Pseudomonas* strains on the seed germination of *A. inebrians* (Table S1). Among these strains, strains with a promotion effect on the seed germination were as follows: St1, St2, St3, St4, St5, St6, St7, St8, St9, St10, St11, St12, St13, St14, St15, St16, St21, St22, St23, St24, St25, St26, St27, St28 and St29, strains with no effect on the seed germination of *A. inebrians* were: St17, St18, St19, St20 and St30, St31, St32, St33 and St34 had inhibition effects on seeds germination (Figure 3, Table S1). In conclusion, different strains of a *Pseudomonas* species had the same impact on *A. inebrians* seed germination.

**FIGURE 4:**
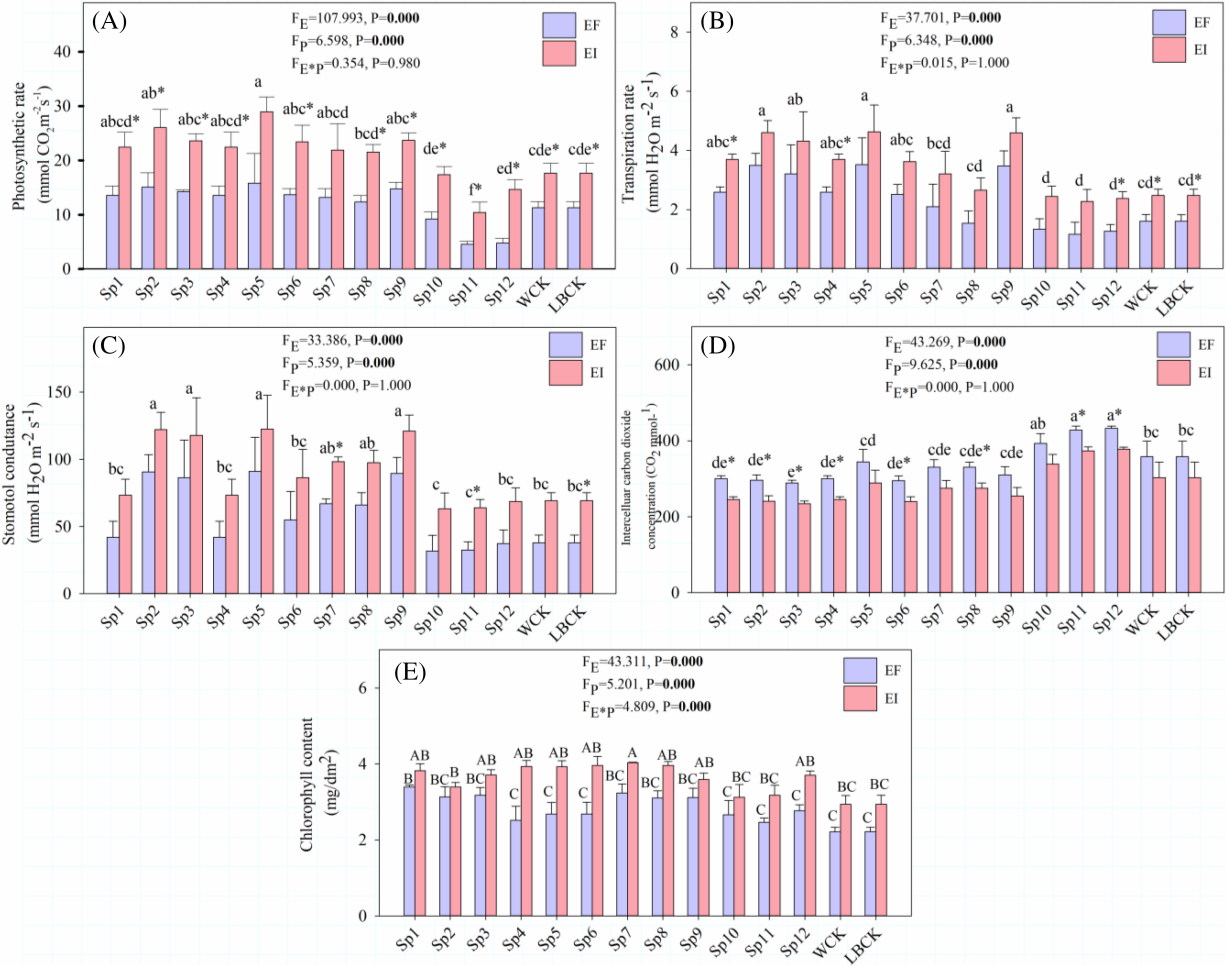
Effects of *Epichloë* endophyte and *Pseudomonas* on *A. inebrians*. Photosynthetic rate (A), transpiration rate (B), stomatol conductance (C), intercelluar carbon dioxide concentration (D) and chlorophyll content (E) of DHG. (EI: Endophyte‐infected, EF: Endophyte‐free, E was the *Epichloë* endophyte, P was inoculating of *Pseudomonas*. Sp1–Sp12 represented inoculating different *Pseudomonas* species. These small letters indicated mean significant difference at *p* < 0.05 among different inoculations. The * represents that there is a significant difference between EI and EF. Different capital letters indicate significant (*p* < 0.05) difference between all treatments.)

Results of the greenhouse pot experiment showed that the *Epichloë* endophyte, inoculating with *Pseudomonas*, and the interaction between the *Epichloë* endophyte and inoculating with *Pseudomonas* had significant (*p* < 0.05) effects on plant length (*F*_E_ = 35.526, *p* = 0.000; *F*_P_ = 5.780, *p* = 0.000; *F*_E*P_ = 2.019, *p* = 0.029); had significant influence on tiller number (*F*_E_ = 7.591, *p* = 0.007; *F*_P_ = 3.746, *p* = 0.000), shoot fresh weight (*F*_E_ = 10.020, *p* = 0.002; *F*_P_ = 5.545, *p* = 0.000), root fresh weight (*F*_E_ = 7.448, *p* = 0.008; *F*_P_ = 6.276, *p* = 0.000), shoot dry weight (*F*_E_ = 11.942, *p* = 0.001; *F*_P_ = 5.011, *p* = 0.000) and root dry weight (*F*_E_ = 6.482, *p* = 0.013; *F*_P_ = 5.080, *p* = 0.000) (Figure S2). Importantly, results showed that inoculating with *Pseudomonas* had significant (*p* < 0.05) effects on the number of reproductive branches in *A. inebrians* (Figure S3). Further, results showed that both the *Epichloë* endophyte and inoculating with *Pseudomonas* had a significant (*p* < 0.05) effect on photosynthetic rate (*F*_E_ = 107.993, *p* = 0.000; *F*_P_ = 6.598, *p* = 0.000), transpiration rate (*F*_E_ = 37.701, *p* = 0.000; *F*_P_ = 6.348, *p* = 0.000), stomatal conductance (*F*_E_ = 33.386, *p* = 0.000; *F*_P_ = 5.359, *p* = 0.000) and intercellular carbon dioxide concentration (*F*_E_ = 43.269, *p* = 0.000; *F*_P_ = 9.625, *p* = 0.000) (Figure 4A–D). The interaction between the *Epichloë* endophyte and inoculating with *Pseudomonas* also had a significant (*p* < 0.05) effect on chlorophyll content (*F*_E_ = 43.311, *p* = 0.000; *F*_P_ = 5.201, *p* = 0.000; *F*_E*P_ = 4.809, *p* = 0.000) (Figure 4E).

The results showed that the *Epichloë* endophyte, inoculating of *Pseudomonas* and the interaction between the *Epichloë* endophyte and inoculating of *Pseudomonas* had a significant (*p* < 0.05) influence on hormone content including IAA (*F*_E_ = 654.055, *p* = 0.000; *F*_P_ = 1234.804, *p* = 0.000; *F*_E*P_ = 604.408, *p* = 0.000), ABA (*F*_E_ = 3199.220, *p* = 0.000; *F*_P_ = 880.359, *p* = 0.000; *F*_E*P_ = 473.657, *p* = 0.000), GA (*F*_E_ = 6.801, *p* = 0.012; *F*_P_ = 1260.580, *p* = 0.000; *F*_E*P_ = 407.476, *p* = 0.000), BR (*F*_E_ = 1381.125, *p* = 0.000; *F*_P_ = 698.652, *p* = 0.000; *F*_E*P_ = 241.534, *p* = 0.000), SA (*F*_E_ = 16.335, *p* = 0.000; *F*_P_ = 770.248, *p* = 0.000; *F*_E*P_ = 779.531, *p* = 0.000), JA (*F*_E_ = 117.082, *p* = 0.000; *F*_P_ = 915.174, *p* = 0.000; *F*_E*P_ = 462.966, *p* = 0.000) and CtK (*F*_E_ = 412.141, *p* = 0.000; *F*_P_ = 385.742, *p* = 0.000; *F*_E*P_ = 225.661, *p* = 0.000) of *A. inebrians* leaves (Figure S4).

The results showed that the *Epichloë* endophyte, inoculating of *Pseudomonas*, and the interaction between the *Epichloë* endophyte and inoculating of *Pseudomonas*, had significant (*p* < 0.05) influence on total nitrogen (*F*_E_ = 168.252, *p* = 0.000; *F*_P_ = 42.879, *p* = 0.000; *F*_E*P_ = 16.116, *p* = 0.000), total phosphorus (*F*_E_ = 286.618, *p* = 0.000; *F*_P_ = 10.293, *p* = 0.000; *F*_E*P_ = 28.097, *p* = 0.000), total potassium (*F*_E_ = 204.796, *p* = 0.000; *F*_P_ = 5.240, *p* = 0.000; *F*_E*P_ = 15.357, *p* = 0.000) and total sodium (*F*_E_ = 609.910, *p* = 0.000; *F*_P_ = 302.200, *p* = 0.000; *F*_E*P_ = 181.522, *p* = 0.000); meanwhile, the *Epichloë* endophyte had a significant (*p* < 0.05) effect on organic carbon (*F*_E_ = 56.790, *p* = 0.000) (Figure S5).

In conclusion, compared with inoculating LB nutrient medium and sterile water, inoculation of Sp10, Sp11 and Sp12 had a significantly negative influence on plant growth and photosynthesis, while inoculation of Sp1, Sp3, Sp5 and Sp7 had a significantly positive effect on plant growth and photosynthesis of *A. inebrians*.

### *Analysis of the multifarious effects of* Pseudomonas *on* A. inebrians *growth via pan‐genome analyses method*

The results showed that the length of the whole genome was Sp1 (6,529,891 bp), Sp3 (5,483,521 bp), Sp5 (4,810,218 bp), Sp7 (5,134,834 bp), Sp10 (5,388,833 bp), Sp11 (6,384,282 bp) and Sp12 (6,089,740 bp) (Table S2). In addition, the GC content of each strain was: Sp1 (*Pseudomonas putida*) (61.81%), Sp3 (*Pseudomonas oryzihabitans*) (61.28%), Sp5 (*Pseudomonas* sp. TF6) (62.43%), Sp7 (*Stutzerimonas stutzeri*) (62.37%), Sp10 (*Pseudononas koreensis*) (60.96%), Sp11 (*Pseudomonas parafulva*) (61.88%) and Sp12 (*Pseudomonas fulva*) (60.94%), respectively (Table S2). Results showed that Sp12 had the highest rRNA genes and tRNA genes number than other bacteria, Sp5 had the highest coding density than other bacteria, and seven bacteria had similar protein‐coding sequence numbers (Table S2). The functional annotation results of COG genes in seven strains mainly included amino acid transport and metabolism, general function prediction, energy production and conservation, inorganic ion transport and metabolism, and transcription (Figure S6).

The results of the horizontal transfer gene prediction of each strain showed that the average value of the horizontal transfer gene of the four growth‐promoting strains was 641, and that of the three inhibitory strains was 719. The number of horizontal transfer genes of the inhibitory strains (Sp10, Sp11 and Sp12) was much higher than that of the growth‐promoting strains (Sp1, Sp3, Sp5 and Sp7). In particular, the number of horizontal transfer genes in the Sp11 strain was as high as 1069 (Table S3). The results of structural variation showed that the number of SNPs and small indels in the Sp11 strain was the least, but the number of structural variations in Sp11 was much higher than that in other strains (Table S4).

**FIGURE 5:**
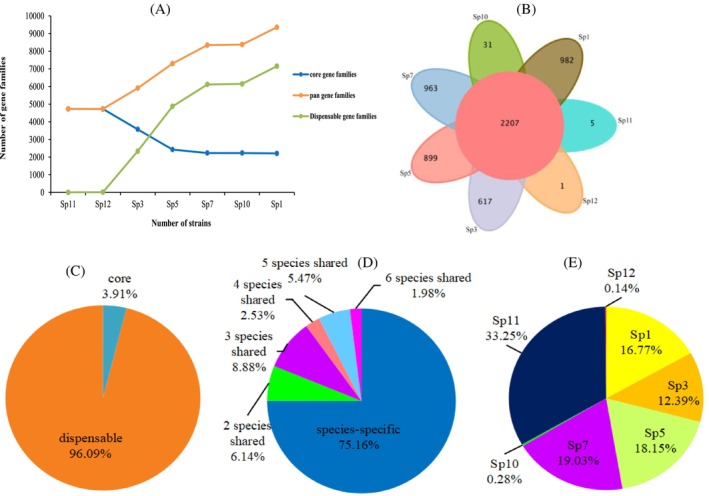
Pan‐genome analyse for *Pseudomonas*. (A) was line chart of the number of gene families; (B) represented petal chart of the number of gene families; the proportion of core genome (C); variable genome of species shared (D) and specific genome (E) of seven strains.

Pan‐genome analysis showed that the number of pan‐gene families of strains Sp1, Sp3, Sp5, Sp7, Sp10, Sp11 and Sp12 was 9362, including 2207 core gene families and 7155 variable gene families (Figure 5A,B). The core genome of seven *Pseudomonas* strains accounted for only 3.91% of the total pan‐genome, and the variable genome accounted for 96.09% of the total pan‐genome of these seven isolates (Figure 5C). An in‐depth analysis of the variable genome showed that 75.16% of the variable genome genes with a length of 18,003,479 bp were unique to the seven isolates (Figure 5D), among which Sp11 accounted for the highest proportion of 33.25% of the unique genes, and Sp12 and Sp10 accounted for less than 0.5% of the unique genes (Figure 5E). KEGG annotation for core genome mainly concentrated on ABC transporters, response‐regulatory receptor domains, LysR substrate binding domains, intima components of binding protein‐dependent transport systems and histidine kinases‐, DNA cyclase B‐, and HSP90‐like ATPase (Figure S7A), and annotation for dispensable genome mainly were LysR substrate binding domains, major facilitation superfamilies, ABC transporters, and binding protein‐dependent transport system cell membrane components (Figure S7B). The annotation information of the unique gene families of the seven strains was different, such as Sp1 and Sp10 possessed the most genes for ABC transporters, and other strains possessed the most genes for two‐component system, but mainly distributed in four categories: ABC transporter, two‐component system, amino acid biosynthesis, and carbon metabolism (Figure S8).

**FIGURE 6:**
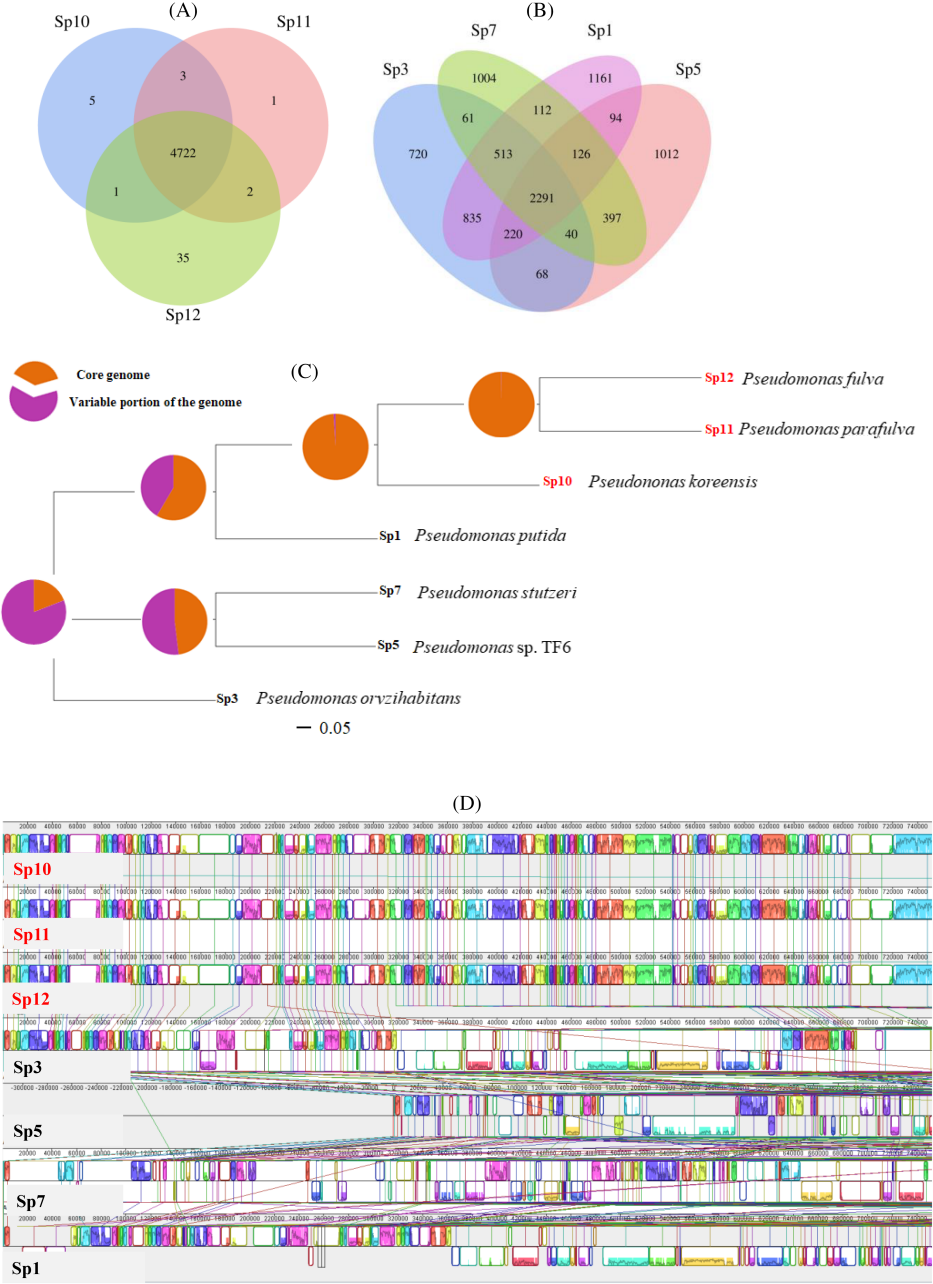
Core‐genome and disable‐genome of strains were analysed. (A) was the number and petal map of the core genomes of Sp10, Sp11 and Sp12. (B) The number and petal map of Sp1, Sp3, Sp5 and Sp7; Phylogenetic tree (C) and collinear graph (D) of each strain.

The effects of *Pseudomonas* strains on *A. inebrians* were divided into two categories: growth promotion (Sp1, Sp3, Sp5 and Sp7) and inhibition (Sp10, Sp11 and Sp12). The influence of different trends of *Pseudomonas* on *A. inebrians* on the distribution of core genes in the genome was studied. The results showed that the number of core genomes of the three inhibitory strains Sp10, Sp11 and Sp12 was 4722, accounting for 99% of the total 4769 pan‐genomic genes of the three strains (Figure 6A), and the number of core genomes of the four promoting strains Sp1, Sp3, Sp5 and Sp7 was 2291. Twenty‐six per cent of 8654 pan‐genomic genes were in the four growth‐promoting strains (Figure 6B). Phylogenetic relationship results showed that Sp10, Sp11 and Sp12 were closely related (Figure 6C). Different lines in the collinearity diagram represent the whole‐genome gene sequence of different strains, and line segments on each line represent different gene segments. Strains Sp10, Sp11 and Sp12 have many of the same gene segments at the same gene locus (Figure 6D). This result indicated that Sp10, Sp11 and Sp12 had high homology, and homology represented the same or a similar function of strains. The above results were consistent with the results of the previous germination test and pot experiment of this study; Sp10, Sp11 and Sp12 had inhibitory effects on *A. inebrians*.

Genome islands were predicted for strains Sp1, Sp3, Sp5, Sp7, Sp10, Sp11 and Sp12 (Table S5); the results showed that three strains Sp10, Sp11 and Sp12 had the same or similar (more than 95% similarity) six gene islands: Genomic_island_1, Genomic_island_2, Genomic_island_3, island_5, island_6 and Genomic_island_8, respectively (Figure S9). The six gene islands contained a total of 151 gene fragments, and none of the above gene islands were found in Sp1, Sp3, Sp5 and Sp7 (Table S5).

The KEGG database was used to annotate gene function information, and biological function annotation of the above gene fragments was conducted. It was found that 52 gene fragments could be annotated into the KEGG database, these genes were annotated to 49 metabolic pathways, which mainly include the transposase IS5 family and 2‐dehydro‐3‐deoxyphosphooctonate aldolase (KDO 8‐P synthase) (Table S6).

## DISCUSSION

The present study analysed the composition and diversity of phyllosphere culturable bacteria associated with leaves of *A. inebrians*, and it was found that the presence of the *Epichloë* endophyte had a significant effect on the composition and diversity of culturable bacteria. The study also found that endophytic bacteria had higher diversity and a higher Chao index than epiphytic bacteria. The 12 isolates of *Pseudomonas* that were used to inoculate seeds and seedlings of *A. inebrian*s were selected from those obtained from the leaves of this study. Their presence and that of the *Epichloë* endophyte had a diversified impact on *A. inebrians* germination and seedling growth, and this diversified impact was related to the differences of *Pseudomonas* gene composition especially their genomic island, notably genomic islands 1, 2, 3, 5, 6 and 8.

### *The effect of* Epichloë *endophyte on the composition and diversity of* A. inebrians *phyllosphere culturable bacteria*

The present study of the phyllosphere of *A. inebrians* found that Proteobacteria was the most abundant phylum, followed by Firmicutes, Bacteroidetes, Actinobacteria and Flavobacteria. Previous studies found that the majority of the bacterial communities of *A. inebrians* belonged to the Firmicutes, Bacterodetes and Actinobacteria using high‐throughput sequencing technology, it also found that Proteobacteria was the most dominant phylum in the phyllosphere (Liu et al., 2022), and of the rhizosphere (Ju et al., 2020), and the seed‐borne (Liang et al., 2023) bacterial communities of *A. inebrians*. Similarly, another study, utilizing traditional isolation and culture techniques, also showed that the four dominant rhizosphere bacterial phyla of *A. inebrians* were Proteobacteria, Firmicutes, Bacteroidetes and Actinobacteria (Ju et al., 2021).

A similar study indicated that the *Epichloë* endophyte increased the diversity of phyllosphere (including epiphytic and endophytic) bacteria in *A. inebrians* (Liu et al., 2022); but another study concluded that the *Epichloë* endophyte had a negligible effect on the phyllosphere endophytic bacterial communities of Schedonorus phoenix (=*Festuca arundinacea*, tall fescue) (Nissinen et al., 2019). Another study showed that loline alkaloids produced by *Epichloë* endophytes can be used by epiphytic bacteria, thus regulating the phyllosphere of epiphytic bacterial communities (Roberts & Lindow, 2014). Previous studies that focused on the influence of the *Epichloë* endophyte of *A. inebrians* on the diversity of bacterial communities of the rhizosphere, such as Ju et al. (2020), found that the presence of the *Epichloë* endophyte decreased the Shannon diversity of the root‐associated bacterial community while increasing the species richness of the rhizosphere cultural bacterial communities (Ju et al., 2021). The presence of the *Epichloë* endophyte decreased the diversity of the seed‐borne microbiota, and the richness of the seed‐borne microbiota was decreased except for the epiphytic fungi of glumes (Liang et al., 2023). The present study analysed epiphytic and endophytic bacteria of *A. inebrians* from the surface and from within the leaves of host plants, respectively, and found that endophytic bacteria had higher diversity and Chao index than the epiphytic bacteria.

Another interesting finding of the current study was that *Pseudomonas* was the most prominent genus of the phyllosphere bacterial community of *A. inebrians*, comprising approximately 50% of bacterial isolates. Previous studies have shown that *Pseudomonas* is abundantly associated with roots of *A. inebrians* (Ju et al., 2020, 2021), and another study found that the presence of *Epichloë* endophyte increased the population of *Pseudomonas* in *Lolium multiforum* (Bastías et al., 2021). The widespread association of this bacterial genus with healthy *A. inebrians* plants makes it likely, as with the beneficial association with the *Epichloë* endophyte, that *Pseudomonas* plays an important role in the fitness and development of this grass.

### *The effect of* Pseudomonas *on seed germination and seedling growth of* A. inebrians

The current study found that inoculating with *Pseudomonas* bacteria could promote the growth and development of *A. inebrians*, including the development of reproductive tillers. Inoculating with *Pseudomonas* bacteria could promote seed germination (Purwaningsih et al., 2019), because *Pseudomonas* bacteria secrete and produce bioactive secondary metabolites to promote plant growth, including hydrogen cyanide, siderophores, 2,4‐diacetylphloroglucinol and various structurally diverse lipopeptides (van der Voort et al., 2015). A previous study found that inoculating *A. inebrians* with *P. aeruginosa* had a positive effect on seed germination of EF and EI *A. inebrians* under salt stress (Ju et al., 2021); similarly, the present study found that inoculating *A. inebrians* with *Pseudomonas* species, except *P. koreensis*, *Pseudomonas parafulva* and *P. fulva*, could promote seed germination.

Photosynthesis is affected by many factors, such as light intensity, water, carbon dioxide concentration, photosynthetic pigments and stomatal conductance (Rozpadek et al., 2015). Previous studies on drought resistance of tall fescue and meadow fescue (*F. pratensis*) have found that the presence of the *Epichloë* endophyte promotes stomatal closure, thereby effectively conserving plant water. Lower stomatal conductance makes plants more resistant and thus more competitive when under drought stress (Elbersen & West, 1996; Malinowski et al., 1997). However, the closure of stomata can also have a negative impact on the growth rate of plants because it reduces the uptake and utilization of CO_2_ (Manzur et al., 2022). The results of the current study showed that the presence of the *Epichloë* endophyte increased the stomatal conductivity of leaves of *A. inebrians*, which was consistent with the results of Cui et al. (2022). Higher stomatal conductance is conducive to CO_2_ exchange and improves photosynthetic efficiency, which is a key determinant of plant growth (Fatichi et al., 2014).

Nutrient elements, such as C, N and P, are components of the organic structure in plants and are involved in enzymatic reactions or energy metabolism and physiological regulation. Carbon assimilation is mainly through photosynthesis (Fatichi et al., 2014). Under different habitat conditions, the presence of an *Epichloë* endophyte increased the photosynthetic capacity and thus the C concentration of high grasses (Xia et al., 2018; Xu et al., 2021); in addition, under drought conditions, the carbon content of EI was higher than that of EF plants (Cui et al., 2022). In this study, it was found that the leaves of EI plants were longer than those of EF plants under the treatment of LB medium inoculation, or *Pseudomonas* inoculation. Therefore, the C content of EI plants was generally higher than EF plants. The results indicated that the presence of the *Epichloë* endophyte increased the photosynthetic capacity of the plant.

Plant hormones play a central role in the integration of various environmental signals and endogenous growth processes, which can regulate all aspects of plant growth and development, as well as plant responses to abiotic and biological stresses (Peleg & Blumwald, 2011). ABA is the most studied plant hormone. Its synthesis is one of the fastest plant responses to abiotic stress (Sreenivasulu et al., 2012). The results of this study showed that under normal conditions, ABA content in EI leaf blades was significantly higher than that in EF leaf blades, indicating that the presence of *Epichloë* endophytic fungi significantly increased ABA content in EI leaf blades, thereby enhancing the ability to cope with environmental conditions. As one of the major types of hormones, IAA is involved in many physiological activities of plants and regulates plant growth and development (Zhao et al., 2021). Previous studies have found that the presence of an *Epichloë* endophyte can increase the content of IAA in host plants, thus promoting growth and development under drought stress (Cui et al., 2022). The results of this study were consistent with those findings in that the IAA content of EI leaf blades was significantly higher than those that were EF, and the presence of the *Epichloë* endophyte promoted the growth and development of the host plants.

### *The diversified effects of* Pseudomonas *on* A. inebrians *growth were related to its gene composition and genomic island*

*Pseudomonas* bacteria isolated from plants mostly had a positive influent on rice growth, development and resist fungal disease (Yang et al., 2023); *Pseudomonas* bacteria can dissolve phosphate to increase plant yield by enhancing phosphorus acquisition and distribution of plants (Liu et al., 2024), *Pseudomonas* bacteria also systematically changed the communities of wheat soil PGPR to increase plant growth (Garrido‐Sanz et al., 2023). However, some *Pseudomonas* strains could enhance the susceptibility of plants to diseases like rice sheath rot (Kim et al., 2015). Researchers found that special and unique genomic composition and a genomic island (lipopeptide/quorum‐sensing, LPQ) of *P. fluorescens* strains was the main reason for the above results (Melnyk et al., 2019), and this finding was consistent with the present study which found that many *Pseudomonas* bacterial strains play a positive role in the growth of *A. inebrians*; however, several *Pseudomonas* strains had a negative effect on the growth of *A. inebrians* and this was linked to the genetic island factor.

Pan‐genomic technology plays an important role in analysing plant evolution, research on the analysis of bacteria based on pan‐genomic technology revealed genomic variations associated with domestication traits of Broomcorn millet (*Panicum miliaceum* L.) (Chen, Liu, et al., 2023), during evolution, intracellular gene transfer of genetic material from plastid and mitochondria to the nucleus continuously reshapes the wheat genome and increases its complexity (Chen, Guo, et al., 2023). In this study, the core genome of seven strains of *Pseudomonas* accounted for 3.91% of the pan‐genome, and the core genome of the four growth‐promoting strains Sp1, Sp3, Sp5 and Sp7 accounted for 26% of the pan‐genome. The core genomes of the Sp10, Sp11 and Sp12, which had inhibitory effects on seed germination and growth, accounted for 99% of the total pan‐genome genes, and the percentage of core genomes was close to 100%.

Apart from vertical transmission of the genome to offspring, bacteria can also access genetic material from the environment through horizontal gene transfer (Soucy et al., 2015). There are several mechanisms by which genes are transferred between bacteria, including active acquisition, gene extraction and gene fragment transfer (Thomas & Nielsen, 2005). Bacteria gene fragments are also frequently lost in the environment (Ding et al., 2018). Freschi et al. (2019) conducted a pan‐genomic analysis of 1311 whole‐genome results of *P. aeruginosa* and found that horizontal gene transfer contributed to the antimicrobial resistance and virulence mechanism of this species in infected humans. Pan‐genomic analyses of *Mycobacterium* have also shown that horizontal gene transfer plays a key role in the evolution of strains to adapt to new habitats and hosts (Dumas et al., 2016). The results of this study showed that the number of horizontal transfer genes of the inhibitory strain 719 was much higher than the number of horizontal transfer genes of the growth‐promoting strain 641, so it was speculated that the transfer of these horizontal genes could promote the transformation of growth‐promoting strains to inhibitory strains. In a subsequent study, assisted gene knockout and gene editing techniques could be considered to verify the transformation.

Genomic island of LPQ (lipopeptide/quorum‐sensing) was a known determinant of pathogenicity in *P. brassicacearum*, *P. corrugata* and *P. mediterranea* (Licciardello et al., 2012), and the T3SS (type III secretion system) island also was found to be involved in suppressing host plant immunity in beneficial rhizosphere strains (Marchi et al., 2013). The T3SS gene island was found only in strains that were beneficial to or symbiotic with plants, and LPQ gene islands were found only in strains that were pathogenic to plants (Melnyk et al., 2019). The present study found that *Pseudomonas* strains that inhibit *A. inebrians* growth contain several specific gene islands, such as Genomic_island_1, that are not present in other strains that promote *A. inebrians* growth. These gene islands are likely to be related to the inhibition effect of the strain on the host plant. In a follow‐up study, the function verification of the above gene islands can be considered by employing gene knockout or gene editing.

In conclusion, the present study separated bacteria from the phyllosphere of *A. inebrians* and found that the presence of the *Epichloë* endophyte in *A. inebrians* significantly influences bacterial communities of the phyllosphere and their diversity and that *Pseudomonas* was the most dominant genus. The testing of 12 selected *Pseudomonas* isolates for their effects on seed germination and seedling growth found that *Pseudomonas* could have significant positive and negative effects on *A. inebrians*. Whole‐genome and pan‐genome results showed that these diversity effects were related to gene compositions and genomic islands of *Pseudomonas* strains. A total of six similar genomic islands were present in inhibitory strains Sp10, Sp11 and Sp12, but these were absent in the other nine strains. Further research involving gene editing and other technologies on these islands is required to investigate the ability to change the inhibition characteristics of Sp10, Sp11 and Sp12.

## AUTHOR CONTRIBUTIONS

**Jinjin Liang:** Conceptualization; investigation; methodology; writing – original draft; writing – review and editing. **Bowen Liu:** Software; data curation. **Michael J. Christensen:** Validation; formal analysis. **Chunjie Li:** Supervision; funding acquisition. **Xingxu Zhang:** Visualization; project administration. **Zhibiao Nan:** Resources; project administration.

## CONFLICT OF INTEREST STATEMENT

The authors declare no conflicts of interest.

## Supporting information


**Data S1.** Supporting Information.

**Table S5.** The id of gene islands predicted by seven strains and the information of gene fragments contained in the gene islands were involved in this paper. (Note: Because the attached table is too large, the title part of the chart, such as strain number, gene island number and gene number, has been marked with orange. The six‐key gene islands with high similarity mentioned in the paper, Sp10, Sp11 and Sp12, have been marked with blue background.)
**Table S6.** Gene function annotation information of gene fragments contained in six gene islands with high similarity in Sp10, Sp11 and Sp12. (Note: Only genes annotated to KEGG function are listed; those that have not been annotated are not shown. As the table is too large, the title parts of the chart, such as strain number, gene island number and gene number, have been marked in orange. ‘✔’ represented a gene present in strain.)

## Data Availability

The data that support the findings of this study are available in the Supporting Information of this article.
