# Supplementary material for: The effects of Pseudomonas strains isolated from Achnatherum inebrians on plant growth: A genomic perspective
Source: Environ Microbiol Rep. 2024 Oct 10;16(5):e70011. doi: 10.1111/1758-2229.70011 (PMC11465459; doi:10.1111/1758-2229.70011)
Supplement: Supplementary file 1 — Data S1. Supporting Information. [file EMI4-16-e70011-s002.pdf]

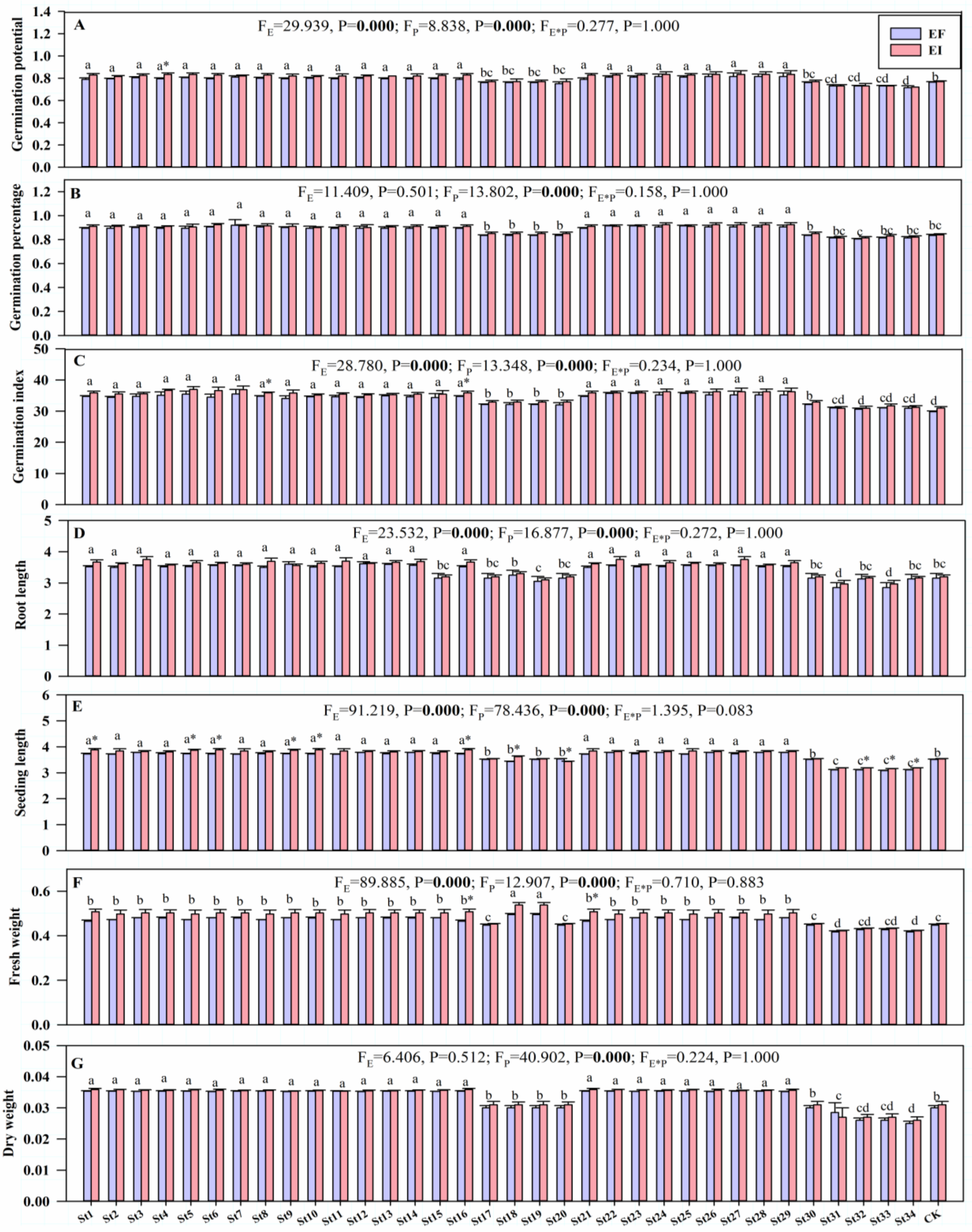

Figure S1. Effects of *Epichloë* endophyte and *Pseudomonas* on germination potential (A), germination percentage (B), germination index (C), root length (D), seeding length (E), fresh weight (F), dry weight (G) of *A. inebrians* seeds. (EI: endophyte-infected, EF: endophyte-free, E was the *Epichloë* endophyte fungi, P was inoculating of *Pseudomonas*. St1-St34 represented inoculating different *Pseudomonas* strains. CK represented inoculating LB fluid nutrient medium on *A. inebrians*. These small letters indicated mean significant difference at P<0.05 among different inoculations. The * represented there is a significant difference between EI and EF.)

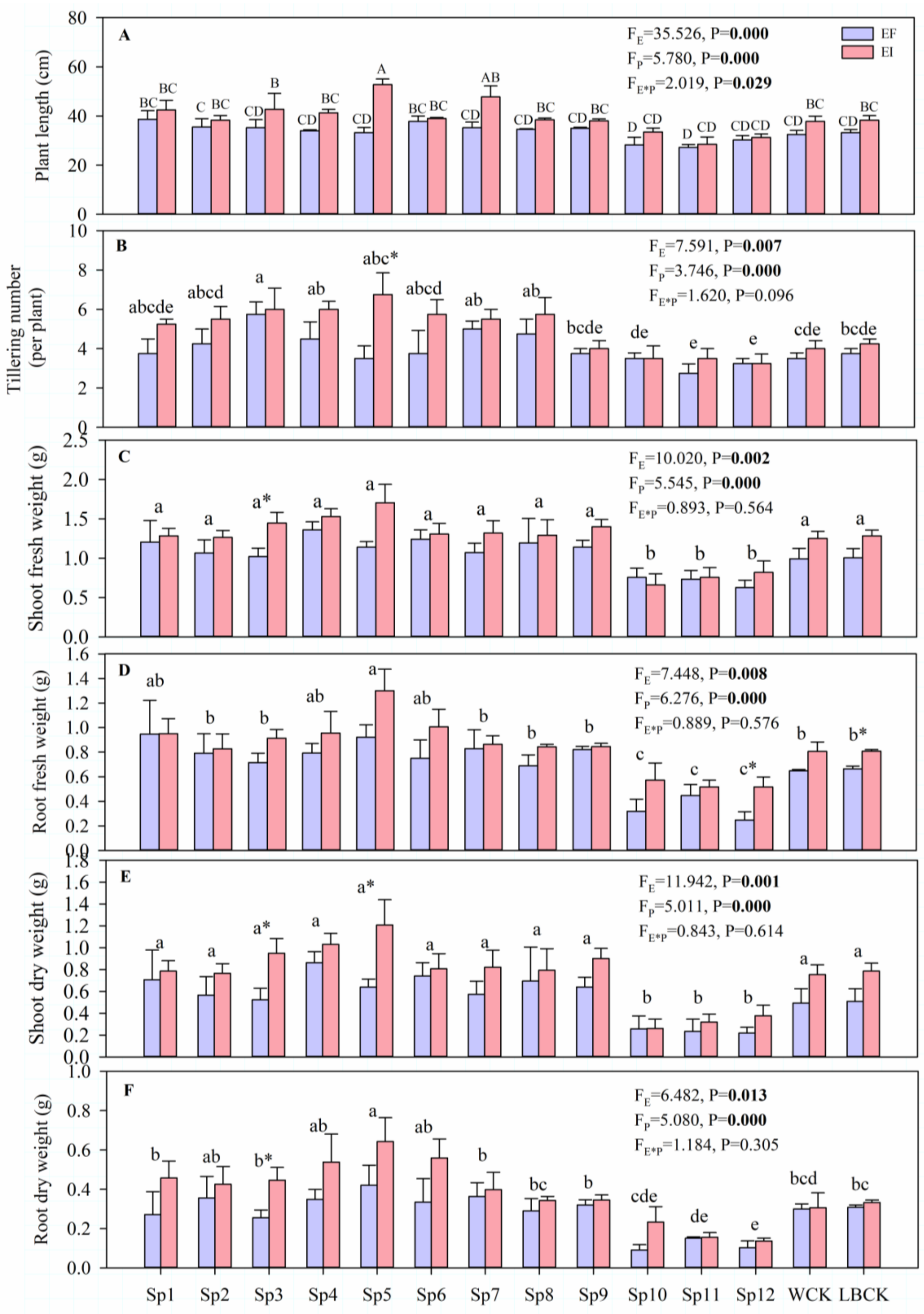

Figure S2. Effects of *Epichloë* endophyte and *Pseudomonas* on plant length (A), tillering number (B), shoot fresh weight (C), root fresh weight (D), shoot dry weight (E) and root dry weight (F) of *A. inebrians* plants. (E was the *Epichloë* endophyte, P was inoculating of *Pseudomonas*. These small letters indicated mean significant difference at P<0.05 among different inoculations of *A. inerbians* plants. The * represented there is a significant difference between EI and EF. Different capital letters indicate significant (P<0.05) difference between all treatments.)

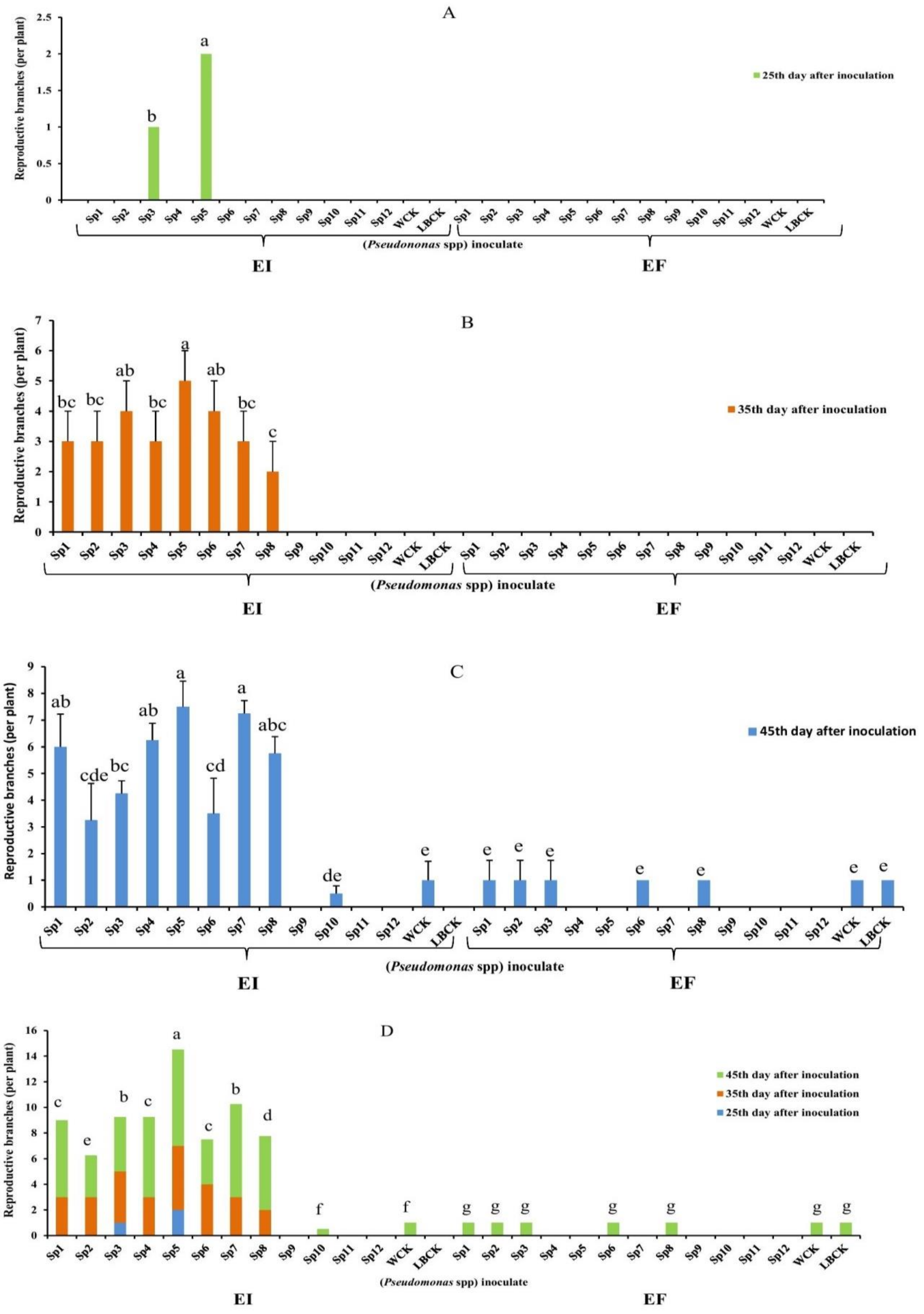

Figure S3. The number of reproductive branches in *A. inebrians* under inoculating *Pseudomonas.* (A) was the number of reproductive branches of *A. inerbains* under inoculating *Pseudomonas* at the 25th day; (B) was the number of reproductive branches of *A. inerbains* under inoculating *Pseudomonas* at the 35th day; (C) was the number of reproductive branches of *A. inerbains* under inoculating *Pseudomonas* at the 45th day; and (D) was the total reproductive branches of *A. inebrians* under inoculating *Pseudomonas*.

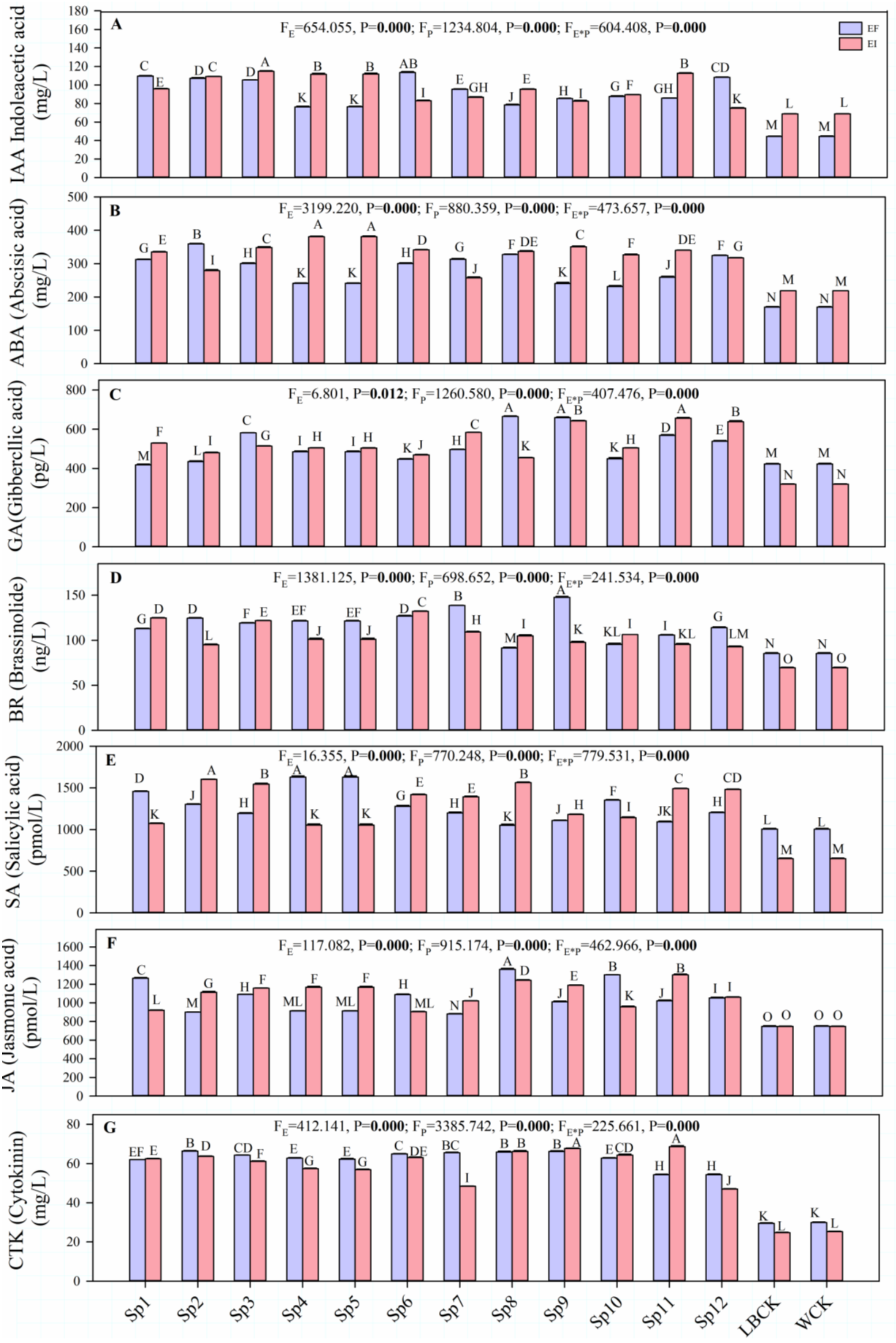

Figure S4. Effects of *Epichloë* endophyte (E) and inoculate *Pseudomonas* strains on IAA (A), ABA (B), GA (C), BR (D), SA (E), JA (F) and BR (G) of *A. inebrians* plants.

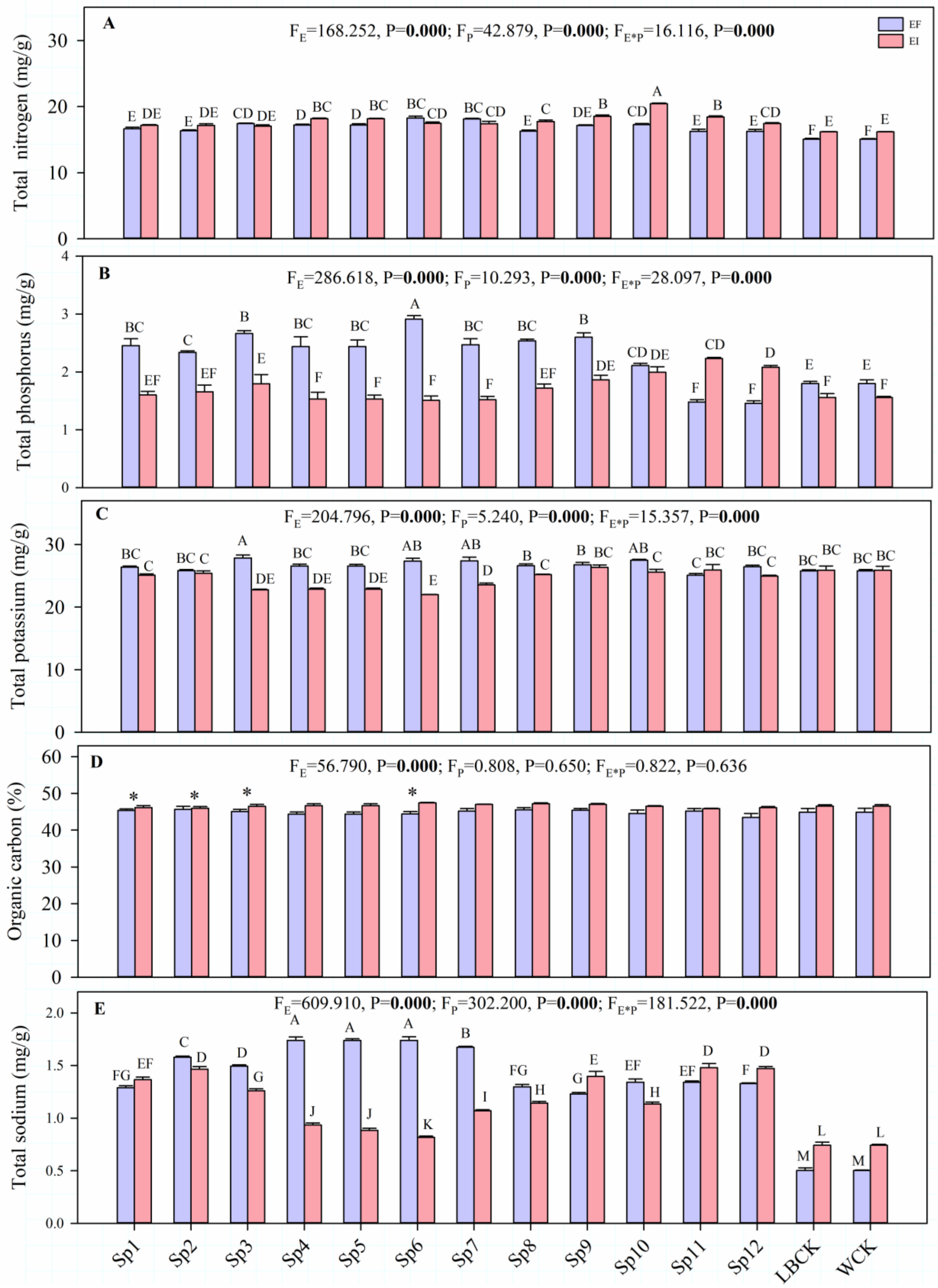

Figure S5. Effects of *Epichloë* endophyte (E) and inoculation of *Pseudomonas* strains on total nitrogen (A), total phosphorus (B), total potassium (C), organic carbon (D) and total sodium (E) of *A. inebrians* plants.

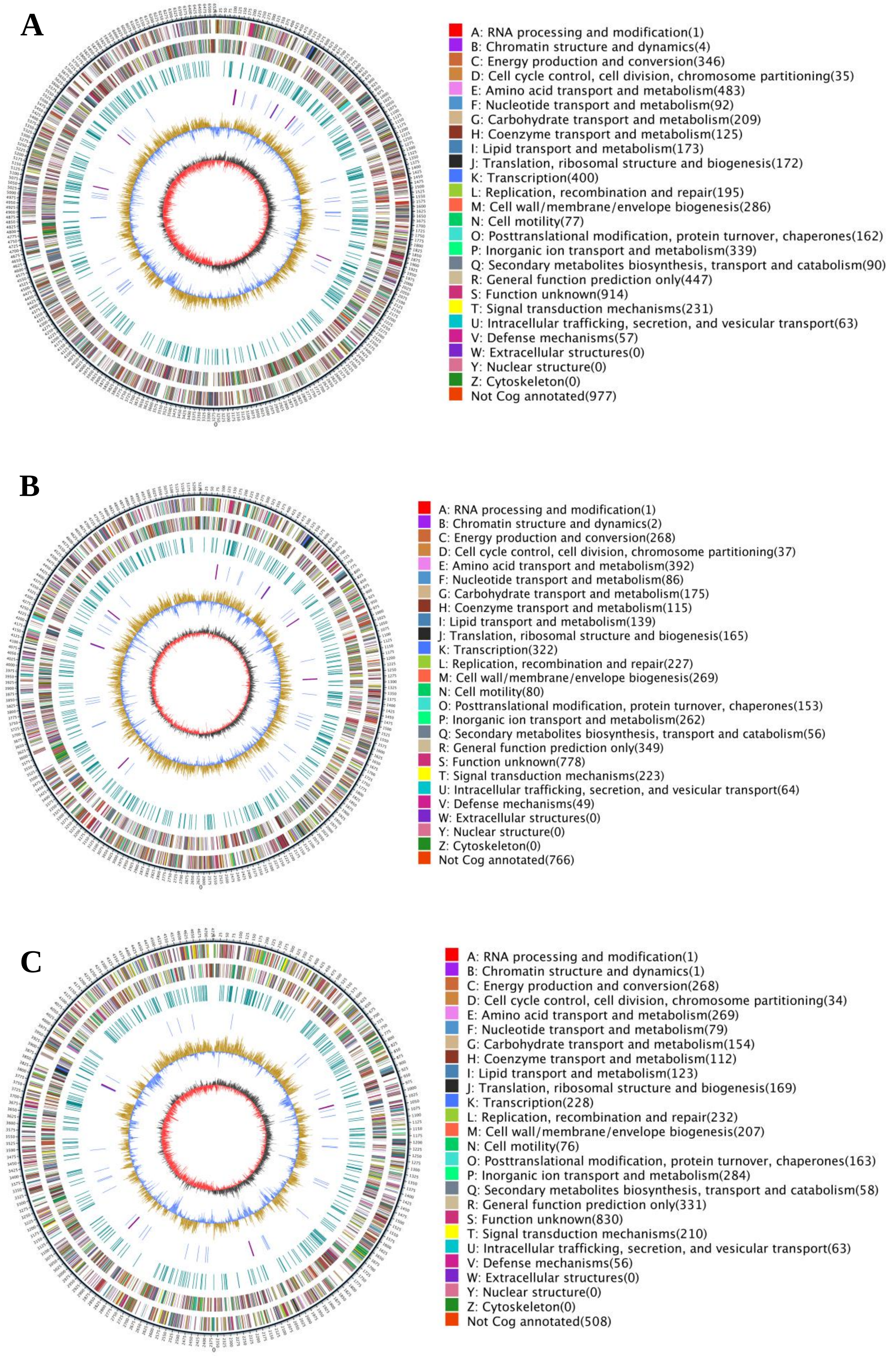
A
A: RNA processing and modification(1)
B: Chromatin structure and dynamics(4)
C: Energy production and conversion(346)
D: Cell cycle control, cell division, chromosome partitioning(35)
E: Amino acid transport and metabolism(483)
F: Nucleotide transport and metabolism(92)
G: Carbohydrate transport and metabolism(209)
H: Coenzyme transport and metabolism(125)
I: Lipid transport and metabolism(173)
J: Translation, ribosomal structure and biogenesis(172)
K: Transcription(400)
L: Replication, recombination and repair(195)
M: Cell wall/membrane/envelope biogenesis(286)
N: Cell motility(77)
O: Posttranslational modification, protein turnover, chaperones(162)
P: Inorganic ion transport and metabolism(339)
Q: Secondary metabolites biosynthesis, transport and catabolism(90)
R: General function prediction only(447)
S: Function unknown(914)
T: Signal transduction mechanisms(231)
U: Intracellular trafficking, secretion, and vesicular transport(63)
V: Defense mechanisms(57)
W: Extracellular structures(0)
Y: Nuclear structure(0)
Z: Cytoskeleton(0)
Not Cog annotated(977)
B
A: RNA processing and modification(1)
B: Chromatin structure and dynamics(2)
C: Energy production and conversion(268)
D: Cell cycle control, cell division, chromosome partitioning(37)
E: Amino acid transport and metabolism(392)
F: Nucleotide transport and metabolism(86)
G: Carbohydrate transport and metabolism(175)
H: Coenzyme transport and metabolism(115)
I: Lipid transport and metabolism(139)
J: Translation, ribosomal structure and biogenesis(165)
K: Transcription(322)
L: Replication, recombination and repair(227)
M: Cell wall/membrane/envelope biogenesis(269)
N: Cell motility(80)
O: Posttranslational modification, protein turnover, chaperones(153)
P: Inorganic ion transport and metabolism(262)
Q: Secondary metabolites biosynthesis, transport and catabolism(56)
R: General function prediction only(349)
S: Function unknown(778)
T: Signal transduction mechanisms(223)
U: Intracellular trafficking, secretion, and vesicular transport(64)
V: Defense mechanisms(49)
W: Extracellular structures(0)
Y: Nuclear structure(0)
Z: Cytoskeleton(0)
Not Cog annotated(766)
C
A: RNA processing and modification(1)
B: Chromatin structure and dynamics(1)
C: Energy production and conversion(268)
D: Cell cycle control, cell division, chromosome partitioning(34)
E: Amino acid transport and metabolism(269)
F: Nucleotide transport and metabolism(79)
G: Carbohydrate transport and metabolism(154)
H: Coenzyme transport and metabolism(112)
I: Lipid transport and metabolism(123)
J: Translation, ribosomal structure and biogenesis(169)
K: Transcription(228)
L: Replication, recombination and repair(232)
M: Cell wall/membrane/envelope biogenesis(207)
N: Cell motility(76)
O: Posttranslational modification, protein turnover, chaperones(163)
P: Inorganic ion transport and metabolism(284)
Q: Secondary metabolites biosynthesis, transport and catabolism(58)
R: General function prediction only(331)
S: Function unknown(830)
T: Signal transduction mechanisms(210)
U: Intracellular trafficking, secretion, and vesicular transport(63)
V: Defense mechanisms(56)
W: Extracellular structures(0)
Y: Nuclear structure(0)
Z: Cytoskeleton(0)
Not Cog annotated(508)

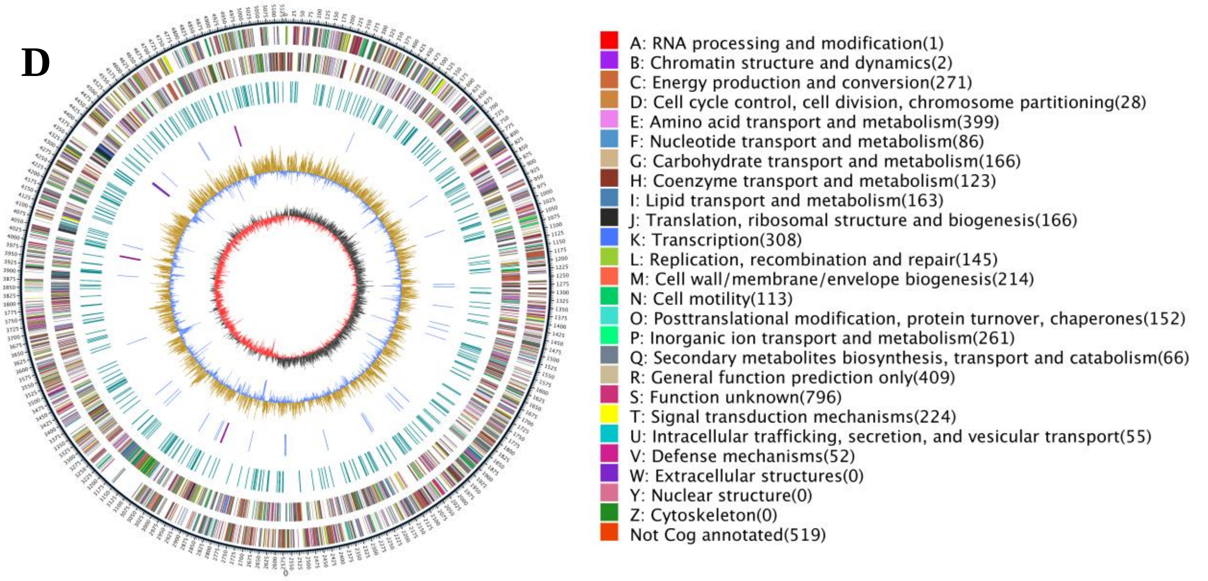
D
A: RNA processing and modification(1)
B: Chromatin structure and dynamics(2)
C: Energy production and conversion(271)
D: Cell cycle control, cell division, chromosome partitioning(28)
E: Amino acid transport and metabolism(399)
F: Nucleotide transport and metabolism(86)
G: Carbohydrate transport and metabolism(166)
H: Coenzyme transport and metabolism(123)
I: Lipid transport and metabolism(163)
J: Translation, ribosomal structure and biogenesis(166)
K: Transcription(308)
L: Replication, recombination and repair(145)
M: Cell wall/membrane/envelope biogenesis(214)
N: Cell motility(113)
O: Posttranslational modification, protein turnover, chaperones(152)
P: Inorganic ion transport and metabolism(261)
Q: Secondary metabolites biosynthesis, transport and catabolism(66)
R: General function prediction only(409)
S: Function unknown(796)
T: Signal transduction mechanisms(224)
U: Intracellular trafficking, secretion, and vesicular transport(55)
V: Defense mechanisms(52)
W: Extracellular structures(0)
Y: Nuclear structure(0)
Z: Cytoskeleton(0)
Not Cog annotated(519)

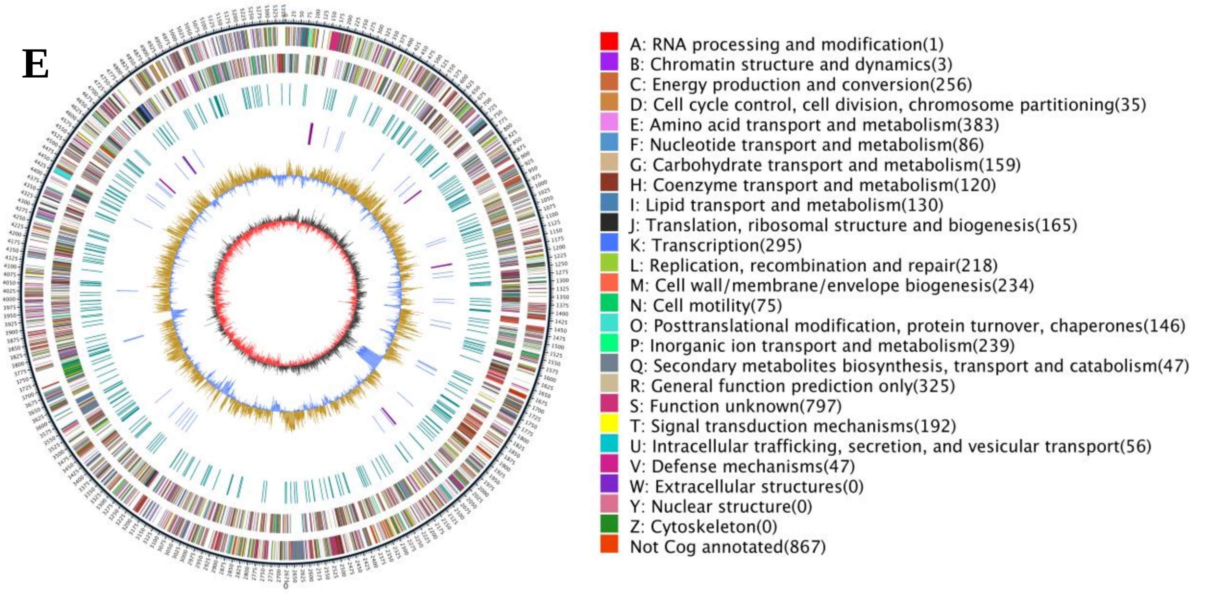
E
A: RNA processing and modification(1)
B: Chromatin structure and dynamics(3)
C: Energy production and conversion(256)
D: Cell cycle control, cell division, chromosome partitioning(35)
E: Amino acid transport and metabolism(383)
F: Nucleotide transport and metabolism(86)
G: Carbohydrate transport and metabolism(159)
H: Coenzyme transport and metabolism(120)
I: Lipid transport and metabolism(130)
J: Translation, ribosomal structure and biogenesis(165)
K: Transcription(295)
L: Replication, recombination and repair(218)
M: Cell wall/membrane/envelope biogenesis(234)
N: Cell motility(75)
O: Posttranslational modification, protein turnover, chaperones(146)
P: Inorganic ion transport and metabolism(239)
Q: Secondary metabolites biosynthesis, transport and catabolism(47)
R: General function prediction only(325)
S: Function unknown(797)
T: Signal transduction mechanisms(192)
U: Intracellular trafficking, secretion, and vesicular transport(56)
V: Defense mechanisms(47)
W: Extracellular structures(0)
Y: Nuclear structure(0)
Z: Cytoskeleton(0)
Not Cog annotated(867)

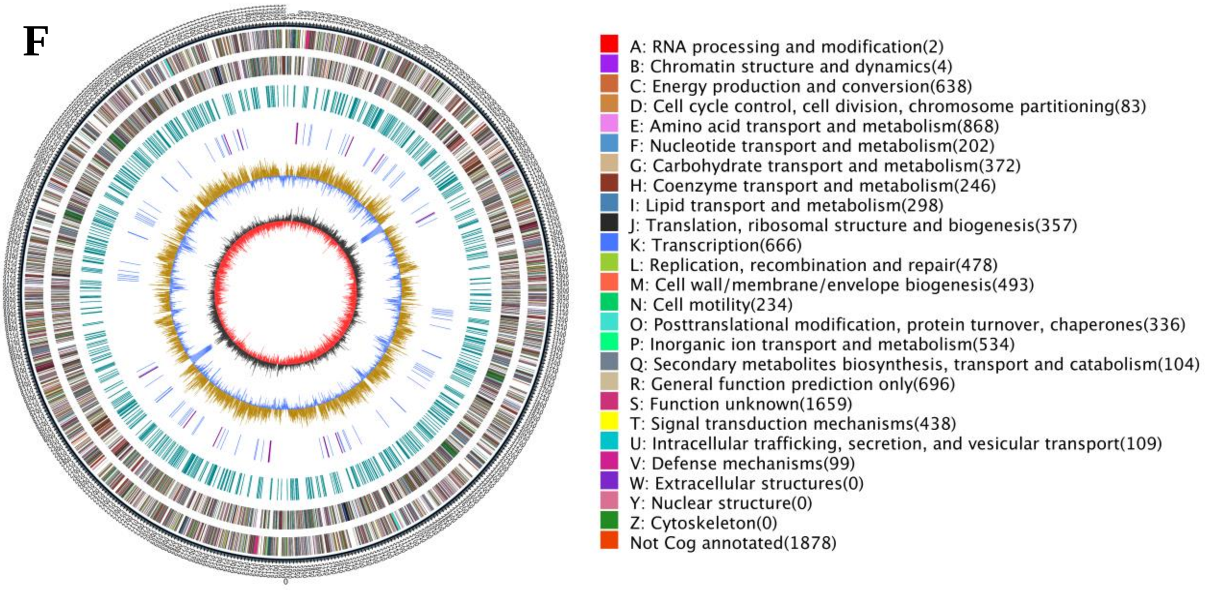
F
A: RNA processing and modification(2)
B: Chromatin structure and dynamics(4)
C: Energy production and conversion(638)
D: Cell cycle control, cell division, chromosome partitioning(83)
E: Amino acid transport and metabolism(868)
F: Nucleotide transport and metabolism(202)
G: Carbohydrate transport and metabolism(372)
H: Coenzyme transport and metabolism(246)
I: Lipid transport and metabolism(298)
J: Translation, ribosomal structure and biogenesis(357)
K: Transcription(666)
L: Replication, recombination and repair(478)
M: Cell wall/membrane/envelope biogenesis(493)
N: Cell motility(234)
O: Posttranslational modification, protein turnover, chaperones(336)
P: Inorganic ion transport and metabolism(534)
Q: Secondary metabolites biosynthesis, transport and catabolism(104)
R: General function prediction only(696)
S: Function unknown(1659)
T: Signal transduction mechanisms(438)
U: Intracellular trafficking, secretion, and vesicular transport(109)
V: Defense mechanisms(99)
W: Extracellular structures(0)
Y: Nuclear structure(0)
Z: Cytoskeleton(0)
Not Cog annotated(1878)

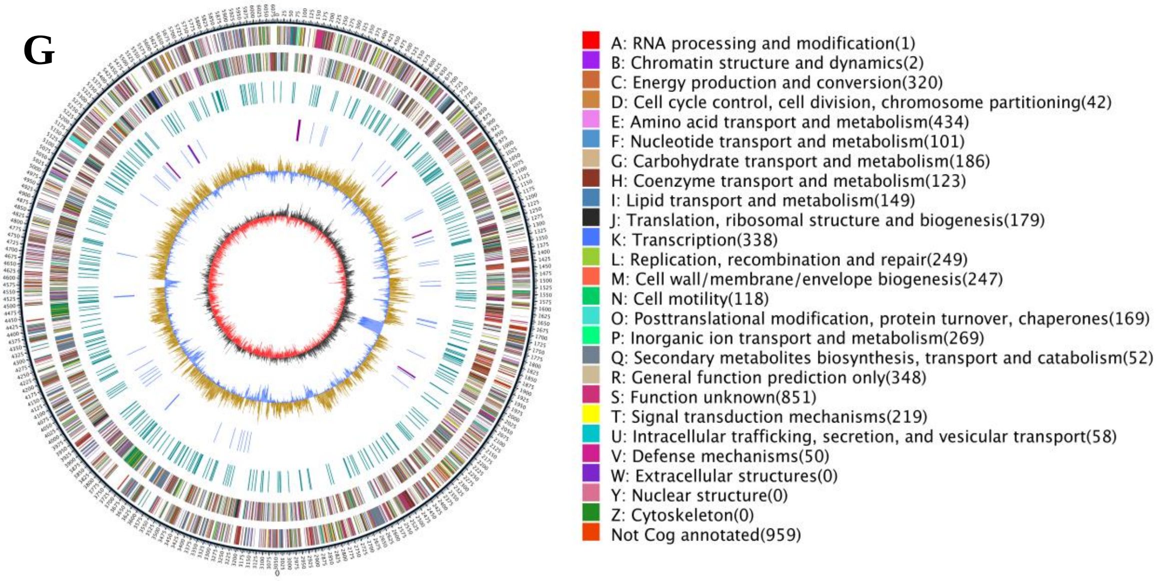

Figure S6. Genomic map of *Pseudomonas*. A: Sp1; B: Sp3; C: Sp5; D: Sp7; E: Sp10; F: Sp11; G: Sp12.

Note: The outermost circle represents the size of the genome, which is 5kb; The second and third circles represent genes on the positive and negative strands of the genome, respectively, with different colors representing different COG functions of genes. The fourth circle is a repeating sequence; the fifth circle has blue tRNA and purple rRNA; The sixth circle represents the GC content. Yellow indicates that the GC content in the region is higher than the average GC content of the genome, while blue indicates that the GC content in the region is lower than the average GC content of the genome. The higher the peak, the greater the difference between the GC content and the average GC content; The innermost circle is GC skew, with red indicating areas where C content is greater than G, and dark gray indicating areas where G content is greater than C.

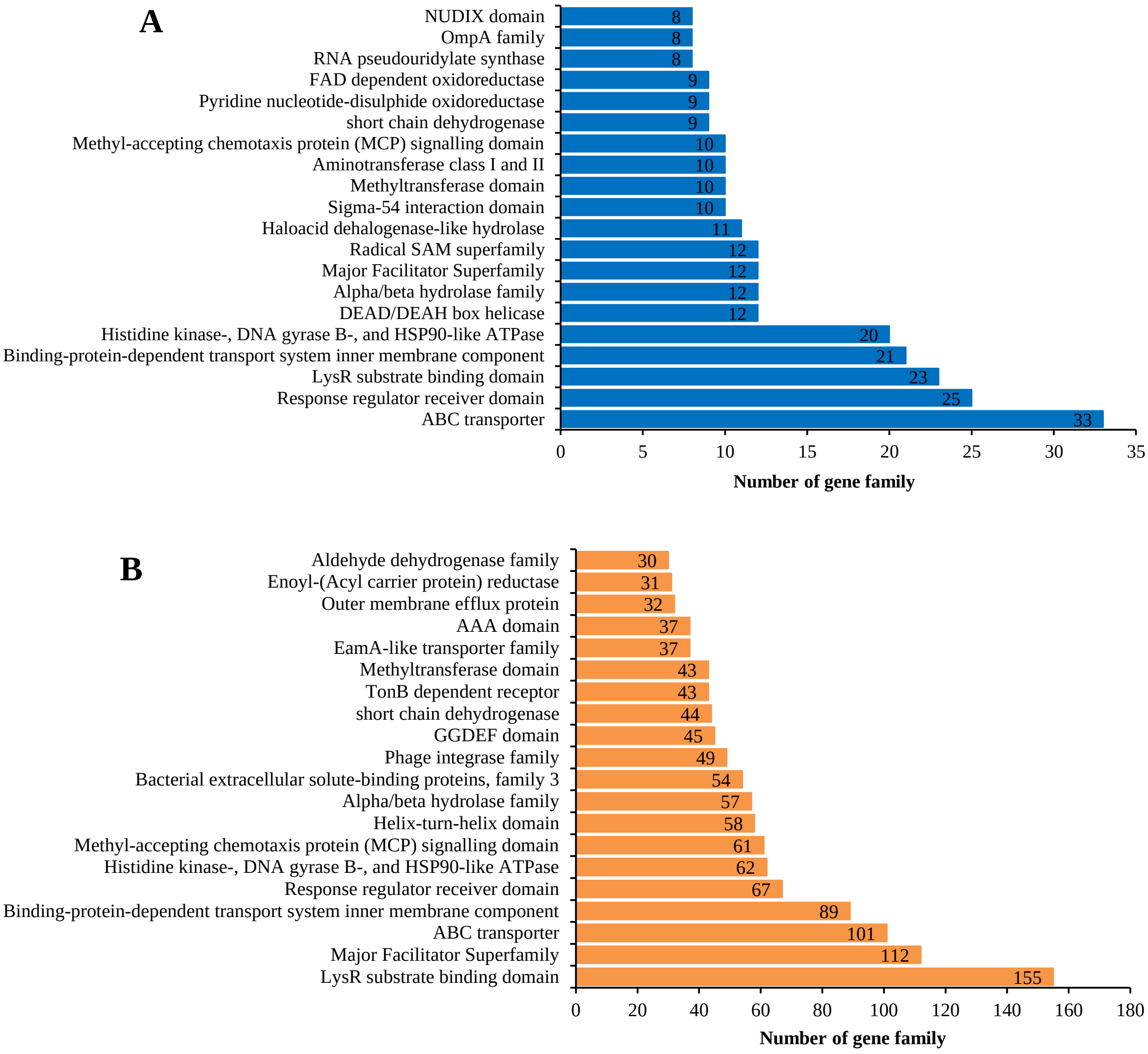

Figure S7. Annotated information of the top 20 core genome (A) and dispensable genome (B).

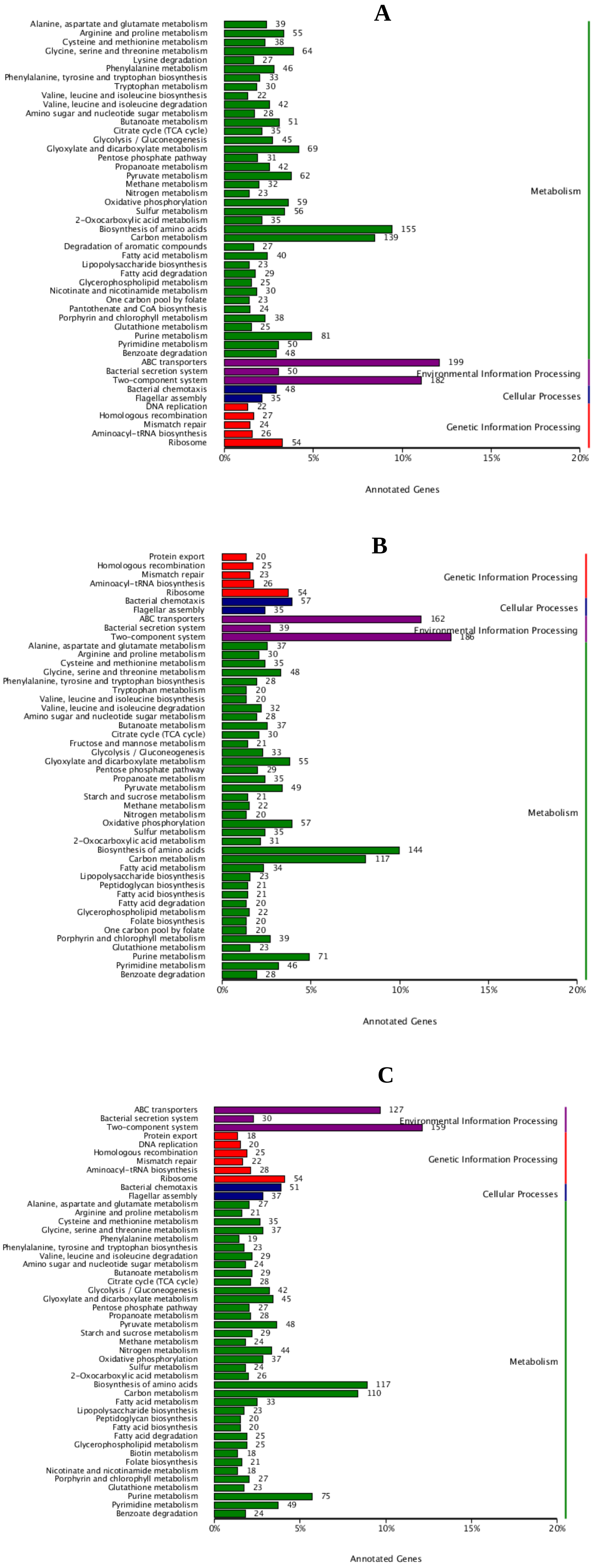

A
Alanine, aspartate and glutamate metabolism 39
Arginine and proline metabolism 55
Cysteine and methionine metabolism 38
Glycine, serine and threonine metabolism 64
Lysine degradation 27
Phenylalanine metabolism 46
Phenylalanine, tyrosine and tryptophan biosynthesis 33
Tryptophan metabolism 30
Valine, leucine and isoleucine biosynthesis 22
Valine, leucine and isoleucine degradation 42
Amino sugar and nucleotide sugar metabolism 28
Butanoate metabolism 51
Citrate cycle (TCA cycle) 35
Glycolysis / Gluconeogenesis 45
Glyoxylate and dicarboxylate metabolism 69
Pentose phosphate pathway 31
Propanoate metabolism 42
Pyruvate metabolism 62
Methane metabolism 32
Nitrogen metabolism 23
Oxidative phosphorylation 59
Sulfur metabolism 56
2-Oxocarboxylic acid metabolism 35
Biosynthesis of amino acids 155
Carbon metabolism 139
Degradation of aromatic compounds 27
Fatty acid metabolism 40
Lipopolysaccharide biosynthesis 23
Fatty acid degradation 29
Glycerophospholipid metabolism 25
Nicotinate and nicotinamide metabolism 30
One carbon pool by folate 23
Pantothenate and CoA biosynthesis 24
Porphyrin and chlorophyll metabolism 38
Glutathione metabolism 25
Purine metabolism 81
Pyrimidine metabolism 50
Benzoate degradation 48
ABC transporters 199
Bacterial secretion system 50
Two-component system 182
Bacterial chemotaxis 48
Flagellar assembly 35
DNA replication 22
Homologous recombination 27
Mismatch repair 24
Aminoacyl-tRNA biosynthesis 26
Ribosome 54
Metabolism
Environmental Information Processing
Cellular Processes
Genetic Information Processing
0%
5%
10%
15%
20%
Annotated Genes
B
Protein export 20
Homologous recombination 25
Mismatch repair 23
Aminoacyl-tRNA biosynthesis 26
Ribosome 54
Bacterial chemotaxis 57
Flagellar assembly 35
ABC transporters 162
Bacterial secretion system 39
Two-component system 186
Alanine, aspartate and glutamate metabolism 37
Arginine and proline metabolism 30
Cysteine and methionine metabolism 35
Glycine, serine and threonine metabolism 48
Phenylalanine, tyrosine and tryptophan biosynthesis 28
Tryptophan metabolism 20
Valine, leucine and isoleucine biosynthesis 20
Valine, leucine and isoleucine degradation 32
Amino sugar and nucleotide sugar metabolism 28
Butanoate metabolism 37
Citrate cycle (TCA cycle) 30
Fructose and mannose metabolism 21
Glycolysis / Gluconeogenesis 33
Glyoxylate and dicarboxylate metabolism 55
Pentose phosphate pathway 29
Propanoate metabolism 35
Pyruvate metabolism 49
Starch and sucrose metabolism 21
Methane metabolism 22
Nitrogen metabolism 20
Oxidative phosphorylation 57
Sulfur metabolism 35
2-Oxocarboxylic acid metabolism 31
Biosynthesis of amino acids 144
Carbon metabolism 117
Fatty acid metabolism 34
Lipopolysaccharide biosynthesis 23
Peptidoglycan biosynthesis 21
Fatty acid biosynthesis 21
Fatty acid degradation 20
Glycerophospholipid metabolism 22
Folate biosynthesis 20
One carbon pool by folate 20
Porphyrin and chlorophyll metabolism 39
Glutathione metabolism 23
Purine metabolism 71
Pyrimidine metabolism 46
Benzoate degradation 28
Genetic Information Processing
Cellular Processes
Environmental Information Processing
Metabolism
0%
5%
10%
15%
20%
Annotated Genes
C
ABC transporters 127
Bacterial secretion system 30
Two-component system 159
Protein export 18
DNA replication 20
Homologous recombination 25
Mismatch repair 22
Aminoacyl-tRNA biosynthesis 28
Ribosome 54
Bacterial chemotaxis 51
Flagellar assembly 37
Alanine, aspartate and glutamate metabolism 27
Arginine and proline metabolism 21
Cysteine and methionine metabolism 35
Glycine, serine and threonine metabolism 37
Phenylalanine metabolism 19
Phenylalanine, tyrosine and tryptophan biosynthesis 23
Valine, leucine and isoleucine degradation 29
Amino sugar and nucleotide sugar metabolism 24
Butanoate metabolism 29
Citrate cycle (TCA cycle) 28
Glycolysis / Gluconeogenesis 42
Glyoxylate and dicarboxylate metabolism 45
Pentose phosphate pathway 27
Propanoate metabolism 28
Pyruvate metabolism 48
Starch and sucrose metabolism 29
Methane metabolism 24
Nitrogen metabolism 44
Oxidative phosphorylation 37
Sulfur metabolism 24
2-Oxocarboxylic acid metabolism 26
Biosynthesis of amino acids 117
Carbon metabolism 110
Fatty acid metabolism 33
Lipopolysaccharide biosynthesis 23
Peptidoglycan biosynthesis 20
Fatty acid biosynthesis 20
Fatty acid degradation 25
Glycerophospholipid metabolism 25
Biotin metabolism 18
Folate biosynthesis 21
Nicotinate and nicotinamide metabolism 18
Porphyrin and chlorophyll metabolism 27
Glutathione metabolism 23
Purine metabolism 75
Pyrimidine metabolism 49
Benzoate degradation 24
Environmental Information Processing
Genetic Information Processing
Cellular Processes
Metabolism
0%
5%
10%
15%
20%
Annotated Genes

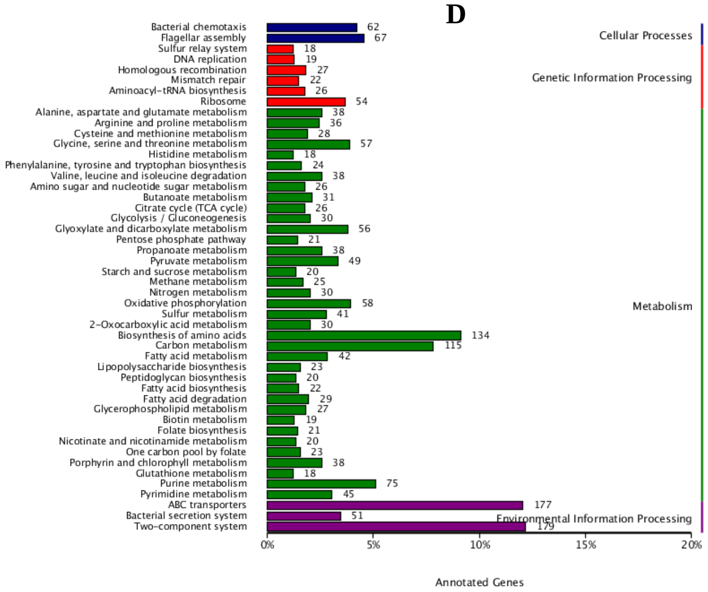
D
Bacterial chemotaxis 62
Flagellar assembly 67
Sulfur relay system 18
DNA replication 19
Homologous recombination 27
Mismatch repair 22
Aminoacyl-tRNA biosynthesis 26
Ribosome 54
Alanine, aspartate and glutamate metabolism 38
Arginine and proline metabolism 36
Cysteine and methionine metabolism 28
Glycine, serine and threonine metabolism 57
Histidine metabolism 18
Phenylalanine, tyrosine and tryptophan biosynthesis 24
Valine, leucine and isoleucine degradation 38
Amino sugar and nucleotide sugar metabolism 26
Butanoate metabolism 31
Citrate cycle (TCA cycle) 26
Glycolysis / Gluconeogenesis 30
Glyoxylate and dicarboxylate metabolism 56
Pentose phosphate pathway 21
Propanoate metabolism 38
Pyruvate metabolism 49
Starch and sucrose metabolism 20
Methane metabolism 25
Nitrogen metabolism 30
Oxidative phosphorylation 58
Sulfur metabolism 41
2-Oxocarboxylic acid metabolism 30
Biosynthesis of amino acids 134
Carbon metabolism 115
Fatty acid metabolism 42
Lipopolysaccharide biosynthesis 23
Peptidoglycan biosynthesis 20
Fatty acid biosynthesis 22
Fatty acid degradation 29
Glycerophospholipid metabolism 27
Biotin metabolism 19
Folate biosynthesis 21
Nicotinate and nicotinamide metabolism 20
One carbon pool by folate 23
Porphyrin and chlorophyll metabolism 38
Glutathione metabolism 18
Purine metabolism 75
Pyrimidine metabolism 45
ABC transporters 177
Bacterial secretion system 51
Two-component system 179
Cellular Processes
Genetic Information Processing
Metabolism
Environmental Information Processing
0% 5% 10% 15% 20%
Annotated Genes

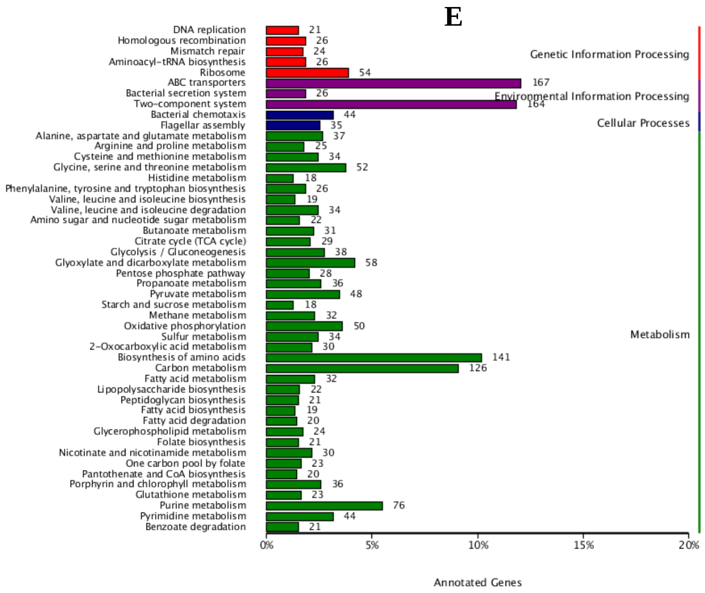
E
DNA replication 21
Homologous recombination 26
Mismatch repair 24
Aminoacyl-tRNA biosynthesis 26
Ribosome 54
ABC transporters 167
Bacterial secretion system 26
Two-component system 164
Bacterial chemotaxis 44
Flagellar assembly 35
Alanine, aspartate and glutamate metabolism 37
Arginine and proline metabolism 25
Cysteine and methionine metabolism 34
Glycine, serine and threonine metabolism 52
Histidine metabolism 18
Phenylalanine, tyrosine and tryptophan biosynthesis 26
Valine, leucine and isoleucine biosynthesis 19
Valine, leucine and isoleucine degradation 34
Amino sugar and nucleotide sugar metabolism 22
Butanoate metabolism 31
Citrate cycle (TCA cycle) 29
Glycolysis / Gluconeogenesis 38
Glyoxylate and dicarboxylate metabolism 58
Pentose phosphate pathway 28
Propanoate metabolism 36
Pyruvate metabolism 48
Starch and sucrose metabolism 18
Methane metabolism 32
Oxidative phosphorylation 50
Sulfur metabolism 34
2-Oxocarboxylic acid metabolism 30
Biosynthesis of amino acids 141
Carbon metabolism 126
Fatty acid metabolism 32
Lipopolysaccharide biosynthesis 22
Peptidoglycan biosynthesis 21
Fatty acid biosynthesis 19
Fatty acid degradation 20
Glycerophospholipid metabolism 24
Folate biosynthesis 21
Nicotinate and nicotinamide metabolism 30
One carbon pool by folate 23
Pantothenate and CoA biosynthesis 20
Porphyrin and chlorophyll metabolism 36
Glutathione metabolism 23
Purine metabolism 76
Pyrimidine metabolism 44
Benzoate degradation 21
Genetic Information Processing
Environmental Information Processing
Cellular Processes
Metabolism
0% 5% 10% 15% 20%
Annotated Genes

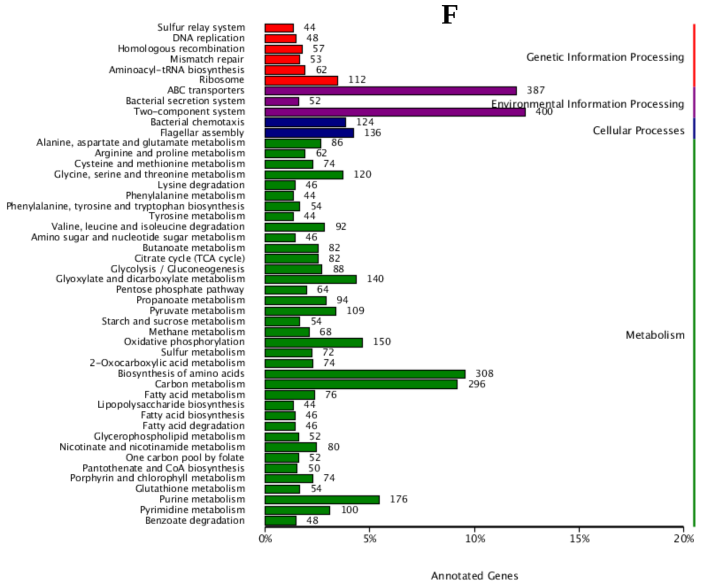
F
Sulfur relay system 44
DNA replication 48
Homologous recombination 57
Mismatch repair 53
Aminoacyl-tRNA biosynthesis 62
Ribosome 112
ABC transporters 387
Bacterial secretion system 52
Two-component system 400
Bacterial chemotaxis 124
Flagellar assembly 136
Alanine, aspartate and glutamate metabolism 86
Arginine and proline metabolism 62
Cysteine and methionine metabolism 74
Glycine, serine and threonine metabolism 120
Lysine degradation 46
Phenylalanine metabolism 44
Phenylalanine, tyrosine and tryptophan biosynthesis 54
Tyrosine metabolism 44
Valine, leucine and isoleucine degradation 92
Amino sugar and nucleotide sugar metabolism 46
Butanoate metabolism 82
Citrate cycle (TCA cycle) 82
Glycolysis / Gluconeogenesis 88
Glyoxylate and dicarboxylate metabolism 140
Pentose phosphate pathway 64
Propanoate metabolism 94
Pyruvate metabolism 109
Starch and sucrose metabolism 54
Methane metabolism 68
Oxidative phosphorylation 150
Sulfur metabolism 72
2-Oxocarboxylic acid metabolism 74
Biosynthesis of amino acids 308
Carbon metabolism 296
Fatty acid metabolism 76
Lipopolysaccharide biosynthesis 44
Fatty acid biosynthesis 46
Fatty acid degradation 46
Glycerophospholipid metabolism 52
Nicotinate and nicotinamide metabolism 80
One carbon pool by folate 52
Pantothenate and CoA biosynthesis 50
Porphyrin and chlorophyll metabolism 74
Glutathione metabolism 54
Purine metabolism 176
Pyrimidine metabolism 100
Benzoate degradation 48
Genetic Information Processing
Environmental Information Processing
Cellular Processes
Metabolism
0% 5% 10% 15% 20%
Annotated Genes

G

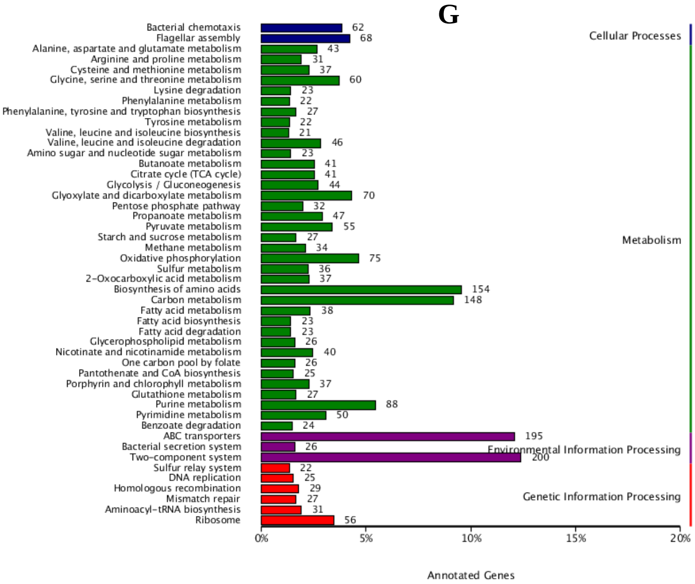

Figure S8. Specific gene family annotation information, A: Strain Sp1; B: Strain Sp3; C: Strain Sp5; D: Strain Sp7; E: Strain Sp10; F: Strain Sp11; G: Strain Sp12.

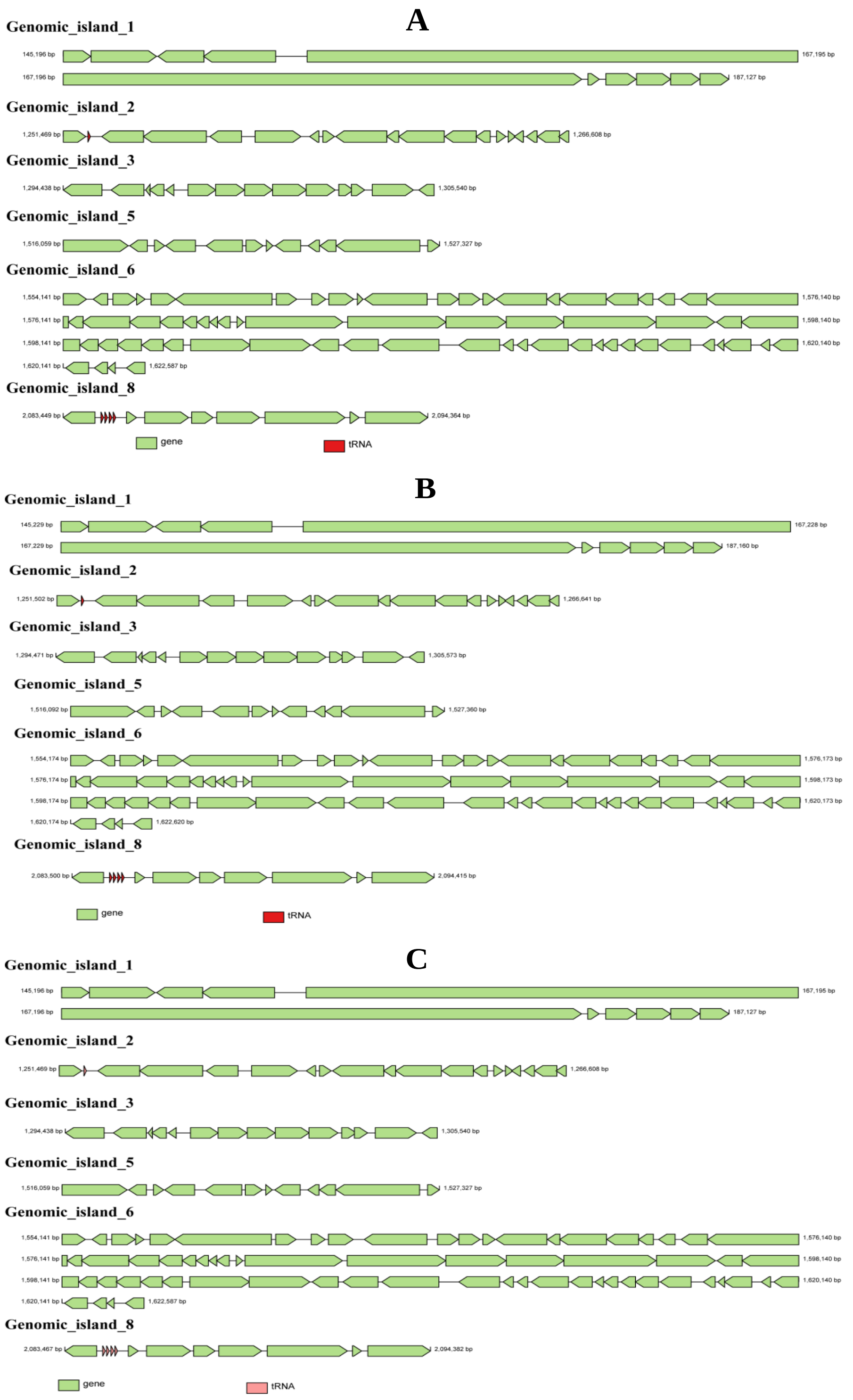

Figure S9. Gene island information shared by three inhibitory strains. (A) represented the gene fragment composition information of the base island in strain Sp10. (B) represented the gene fragment composition information of gene island in strain Sp11. (C) represented the composition information of gene island fragment in strain Sp12.

Table S1. Effects of inoculation of *Pseudomonas* on seed germination and comprehensive evaluation of the growth promotion.

Strains	Mean (U)	comprehensive assessment	Strains	Mean (U)	comprehensive assessment
St1	0.67±0.03ab	P	St18	0.47±0.03c	N
St2	0.65±0.02ab	P	St19	0.45±0.02cd	N
St3	0.68±0.02ab	P	St20	0.39±0.02d	N
St4	0.68±0.02ab	P	St21	0.66±0.02ab	P
St5	0.69±0.03ab	P	St22	0.7±0.02ab	P
St6	0.69±0.03ab	P	St23	0.68±0.01ab	P
St7	0.7±0.04ab	P	St24	0.7±0.03a	P
St8	0.67±0.02ab	P	St25	0.69±0.02ab	P
St9	0.67±0.02ab	P	St26	0.7±0.02ab	P
St10	0.66±0.02ab	P	St27	0.7±0.04a	P
St11	0.66±0.02ab	P	St28	0.69±0.02ab	P
St12	0.66±0.02ab	P	St29	0.7±0.03a	P
St13	0.67±0.02ab	P	St30	0.4±0.01d	N
St14	0.67±0.02ab	P	St31	0.2±0.02e	I
St15	0.62±0.02b	P	St32	0.22±0.02e	I
St16	0.67±0.03ab	P	St33	0.21±0.02e	I
St17	0.4±0.01d	N	St34	0.21±0.01e	I

Note: St1-St34 represented strains of *Pseudomonas*, P was promotion, N was no effect, I was Inhibition. Values were mean ± standard error (SE), n=4 with bars indicating SE. These different small letters represented that there was a significant difference among the 34 inoculated strains at $P<0.05$.

Table S2. Information of *Pseudomonas* strains, based on the genome sequencing

Strain	Genome size (bp)	GC content	rRNA genes	tRNA genes	Coding dentisy (%)	Protein coding sequences (CDS)
Sp1	6529891	61.81%	22	80	88.52%	983
Sp3	5483521	61.28%	22	77	89.59%	986
Sp5	4810218	62.43%	12	62	93.34%	962
Sp7	5134834	62.37%	12	67	89.88%	978
Sp10	5388833	60.96%	22	81	88.64%	979
Sp11	6384282	61.88%	22	78	88.63%	989
Sp12	6089740	60.94%	44	179	88.60%	987

Table S3. Information of horizontal gene transfer

Sample	Share number	Total number
Sp1	0	686
Sp3	0	725
Sp5	0	768
Sp7	0	386
Sp10	0	532
Sp11	0	1069
Sp12	0	555

Table S4. Information of SNP (single nucleotide variation), Small InDel and structural variation

Sample	SNP Number	Small InDel Number	structural variation	
			Insertion Number	Deletion Number
Sp3	359094	15591	14	59
Sp5	86323	4143	1	2
Sp7	126108	6228	1	6
Sp10	338213	11336	2	64
Sp11	256	5	6	144
Sp12	292103	9697	3	72

Note: SNP: nucleic acid variation at a single site; Small InDel: Insertion or deletion of a sequence of small fragments at a location in the genome, less than 50 bp in length; SV: Large gene segment insertion, deletion, repetition, inversion and the length is less than 50 bp.

Table S5. The id of gene islands predicted by 7 strains and the information of gene fragments contained in the gene islands were involved in this paper. (Note: Because the attached table is too large, the title part of the chart, such as strain number, gene island number and gene number, has been marked with orange. The 6 key gene islands with high similarity mentioned in the paper, Sp10, Sp11 and Sp12, have been marked with blue background.)

Table S6. Gene function annotation information of gene fragments contained in 6 gene islands with high similarity in Sp10, Sp11 and Sp12. (Note: Only genes annotated to KEGG function are listed; those that have not been annotated are not shown. As the table is too large, the title parts of the chart such as strain number, gene island number and gene number have been marked in orange. "✔"represented a gene is present in strain.
